# What do the clinical features of positive nontuberculous mycobacteria isolates from patients with HIV/AIDS in China reveal? A systematic review and meta-analysis

**DOI:** 10.7189/jogh.13.04093

**Published:** 2023-09-01

**Authors:** Jianwei Yuan, Yan Wang, Lin Wang, Hongxia Wang, Yuan Ren, Wenzhe Yang

**Affiliations:** 1 Department of Infection, Third Hospital of Shanxi Medical University, Shanxi Bethune Hospital, Shanxi Academy of Medical Sciences, Tongji Shanxi Hospital, Taiyuan, China; 2 School of Public Health, Shanxi Medical University, Taiyuan, China; 3 Department of Epidemiology and Biostatistics, School of Public Health, Tianjin Medical University, Tianjin, China

## Abstract

**Background:**

China has a high burden of nontuberculous mycobacterial (NTM) infections. Immunocompromised populations, such as those with human immunodeficiency virus/acquired immunodeficiency syndrome (HIV/AIDS), are at a higher risk of being infected with NTM than immunocompetent individuals. Yet, there is a paucity of information on the clinical features of positive NTM isolates from patients with HIV/AIDS in China. To address this gap, we conducted a systematic review and meta-analysis of existing studies, comparing them against current expert consensus to provide guidance for clinical practice.

**Methods:**

Two researchers independently searched eight databases (SinoMed, China National Knowledge Infrastructure, Wanfang, VIP, Cochrane Library, PubMed, Embase, and Web of Science) from inception to 26 December 2022 to retrieve published Chinese- and English-language studies reporting clinical features of NTM-positive isolates among patients with HIV/AIDS in China.

**Results:**

We included 28 studies with 1861 patients. The rate of positive NTM isolates detected from men among all patients was 87.3%. NTM species distribution was mainly *Mycobacterium avium* complex (64.3%), which was predominant in different regions. The five most common clinical symptoms were fever (68.5%), cough or expectoration (67.0%), appetite loss (49.4%), weight loss (45.5%), and superficial lymphadenectasis (41.1%). The prevalence of laboratory tests were as follows: albumin <35 g/L (55.6%), erythrocyte sedimentation rate >20 mm/h (91.4%), anaemia (59.0%), predominantly mild, CD4+ T cell count ≤50 pieces/μL (70.3%), and CD4+ T cell count 51-200 pieces/μL (22.1%). Lesion manifestations in thoracic imaging mainly included bilateral lung involvement (83.8%), showed stripe shadows (60.3%), patchy shadows (42.9%), nodules (40.6%), and bronchiectasis (38.6%). Accompanied signs included thoracic lymph node enlargement (49.5%). Seventy per cent of symptoms improved after treatment.

**Conclusions:**

Focusing on clinical symptoms, laboratory tests, and thoracic imaging helps with initial screening for NTM infections. Physicians should raise awareness of the diagnosis and treatment of *Mycobacterium avium* complex, providing guidance for experimental treatment, screening of priority populations for NTM infections, and prophylactic treatment of NTM disease.

**Registration:**

PROSPERO CRD42023388185.

Human immunodeficiency virus/acquired immune deficiency syndrome (HIV/AIDS) is a major contributor to the global burden of disease, accounting for the second highest number of disability-adjusted life years among 10-24, and 25-49-year-olds [1]. At least one million people are currently living with HIV/AIDS in China, with a growing number of reported cases across all age groups [2]. Simultaneously, antiretroviral therapy (ART) has led to a reduction in morbidity and mortality, a gradual increase in average life expectancy, and a decrease in associated opportunistic infections (OIs) in patients with HIV/AIDS [3].

However, the rapid increase in CD6+ T-cell counts during the first three to four months of ART treatment may promote the development of OIs [4]. In high-income countries, including the USA and Canada, disseminated *Mycobacterium avium* complex or *Mycobacterium kansasii* infection of the species nontuberculous mycobacterial (NTM) is the third most common OI, after *pneumocystis jirovecii* pneumonia and oesophageal candidiasis [5]. Moreover, people living with HIV with NTM disease were associated with a long-term case-fatality rate (CFR), with overall CFR increasing from 15.7% at one year to 22.6% at five years [6].

In low- and middle-income countries (LMICs), NTM infections are largely overlooked due to limitations in medical resources and technology, with the first case of NTM lung disease in Ecuador being diagnosed in 2017 [7]. There is also a significant delay in the diagnosis of NTM diseases, especially in rural areas [8], and a high risk of misdiagnosis of *Mycobacterium tuberculosis* (MTB) infections, with a misdiagnosis rate of 92.81% and a maximum misdiagnosis time of 21 years [9]. NTM infections are not a notifiable infectious disease in most countries, and these factors combine to make NTM infections uncommon in studies of OIs in patients with HIV/AIDS in LMICs [10,11].

In fact, the number of NTM infections in LMICs is grossly underestimated. A national survey of tuberculosis (TB) prevalence among participants aged ≥15 years in Gambia showed an NTM separation rate of 39.8% [12] and 29.0% in India [13]. The National TB Epidemiological Sample Survey in China showed that the prevalence rate of NTM isolation increased from 11.1% in 2001 to 22.9% in 2010 [14,15]. The rate of NTM isolation of patients with HIV/AIDS in Shanghai was much higher than suggested by the National TB Epidemiological Sample Survey [16]. Some MTB infections have co-infection with NTM, particularly among patients with HIV [17-19]. NTM infections are also one of the common opportunistic infections in Chinese patients with HIV/AIDS [20-22].

NTM refers to mycobacterial species other than MTB complex and *Mycobacterium leprae* [23], which are commonly found in the natural environment (eg, water and soil) [24,25] and cause infection in susceptible individuals with underlying diseases, including chronic obstructive pulmonary disease, immunodeficiency, and HIV infection [26]. Various NTM infections, which do not have a specific clinical presentation [27], are less susceptible to standard anti-tuberculous drug regimens and require longer treatment durations than MTB infections [28,29].

Currently, there is little information on the clinical features of NTM isolates from patients with HIV/AIDS, which can easily lead to misdiagnosis, underdiagnosis, and even delay in clinical treatment. An expert consensus on diagnosis and treatment of patients with HIV/AIDS combined with NTM infections was published in China only in 2019 [30] and has not been updated since. Moreover, it was based on small sample studies and was unsupported by a systematic review and meta-analysis, the highest level of evidence in evidence-based medicine. Therefore, studying NTM isolates from patients with HIV/AIDS is of great significance not only for China, but for LMICs at large. Accordingly, we conducted a systematic review and meta-analysis of positive NTM isolates from patients with HIV/AIDS in China in terms of gender distribution, species distribution, clinical symptoms, laboratory tests, thoracic imaging manifestations, and treatment outcome.

## METHODS

We followed the Preferred Reporting Items for Systematic Reviews and Meta-Analyses (PRISMA) reporting guidelines in conducting this study [31-33].

### Search strategy

We systematically searched Chinese (SinoMed, China National Knowledge Infrastructure (CNKI), Wanfang, and VIP) and English databases (Cochrane Library, PubMed, Embase, and Web of Science). To capture all relevant literature, we used a combination of subject terms and free terms such as “HIV”, “AIDS”, and “NTM”, adjusted for each database (Table S2 in the **Online Supplementary Document**). We set no restrictions on the type of published literature and limited the time span from inception to 26 December 2022. We searched the included studies’ references for potentially relevant information.

### Inclusion and exclusion criteria

We included cross-sectional, case-control, cohort, or case series studies on Chinese patients with HIV/AIDS associated with positive NTM isolates. The observed indicators were clinical symptoms, laboratory tests, thoracic imaging manifestations, species distribution and treatment outcome. If multiple articles examined a clinical indicators, but were based on the same sample data (e.g. data from the same institution or from the same study period), we only included the article with the most descriptive statistics of the data in question. All articles had to meet the diagnostic criteria for NTM isolated from culture [23].

The exclusion criteria were as follows: non-Chinese and -English literature; duplicate publications; unavailability of the required data; case studies, reviews, book chapters, expert opinions, comments, and so on; and basic studies, such as cellular and animal studies.

### Study selection

Two researchers (L Wang and HX Wang) independently searched the literature, conducted deduplication, and performed the initial title/abstract screening, followed by a full-text screening of the retrieved studies. Subsequently, they collected the relevant data and cross-checked for the appropriateness of inclusion. Disagreements were resolved through discussion or negotiation with a third researcher (Y Ren).

### Quality assessment

As we included studies with varying designs, we used a different risk of bias tools to assess possible sources of bias, depending on the design in question. We used the scales recommended by the Agency for Healthcare Research and Quality (AHRQ) [34] to assess the quality of cross-sectional studies, the Newcastle-Ottawa Scale (NOS) for case-control and cohort studies [35], and the Joanna Briggs Institute (JBI) Critical Appraisal Checklist [36] for case-series studies (Table S3-S6 in the **Online Supplementary Document**). Two investigators (L Wang and HX Wang) independently evaluated the risk of bias in the included studies and cross-checked the results with a third investigator (Y Ren), resolving disagreements through discussion. We tabulated data from the included studies to identify bias in the quality evaluation phase.

### Data extraction

We extracted the following information: first author, publication date, region of study subjects, sample size, study type, and gender distribution, observation indicators (clinical symptoms, laboratory tests, thoracic imaging manifestations, species distribution, and treatment outcome), and key elements of risk of bias evaluation.

### Statistical analysis

Based on clinical considerations, we pooled similar and appropriate characteristics. We performed meta-analyses using Stata, version 17.0 (StataCorp, College Station, Texas, USA) for observations with three or more included studies. We conducted Freeman-Tukey double inverse sine transformation for dichotomous variables with extreme rates (r) of 0 or 1 [37]. We converted continuous variables from medians and quartiles to means and standard deviations according to Luo et al. (online calculator: https://www.math.hkbu.edu.hk/~tongt/papers/median2mean.html) [38], with effect scale mean deviation values/event rates (R values) and 95% confidence intervals (CIs) as effect size indicators. We performed meta-analyses using a fixed-effects model when *I*^2^ was <50% and *P* was ≥0.10 (indicating no statistical heterogeneity in the literature); if *I*^2^ was ≥50% or *P* was ≤0.10 (indicating statistical heterogeneity), we used a random-effects model. Additionally, we performed subgroup analyses of regions and sample sizes to explore the sources of heterogeneity. We considered differences statistically significant at *P* < 0.05.

## RESULTS

### Search results

**Figure 1:**
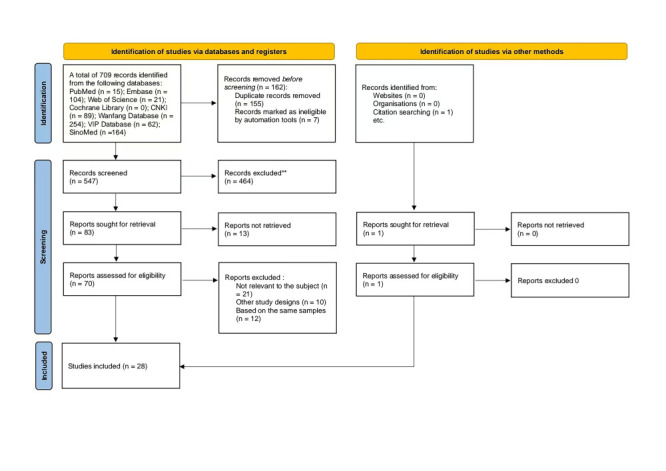
Flowchart of positive NTM in patients with HIV/AIDS systematic review study selection.

We retrieved 709 studies from the preliminary search, including 140 studies in English and 569 in Chinese. After the screening process, we included 28 studies (**Figure 1**).

### Characteristics and quality assessment of included studies

**Figure 2:**
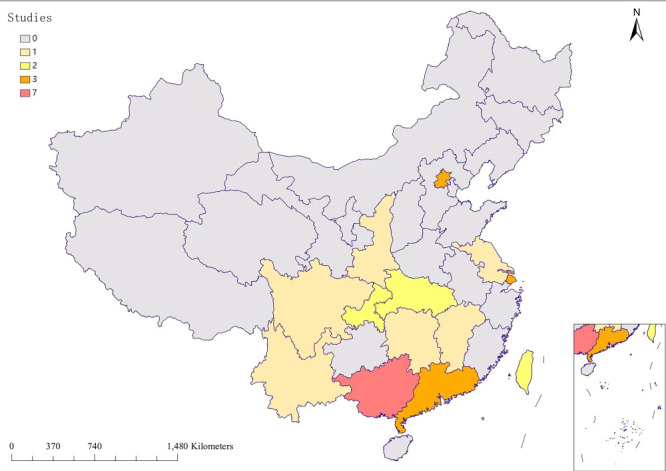
China maps for distribution of included studies.

**Figure 3:**
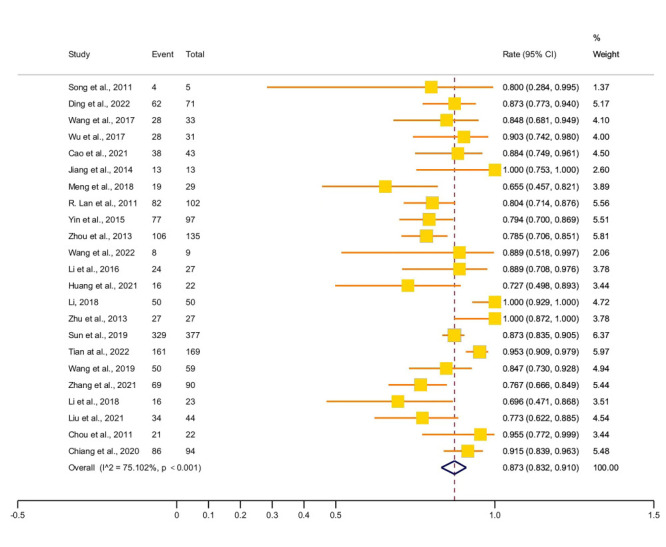
Forest plot of the proportion of men among positive NTM isolates from patients with HIV/AIDS.

Twenty-eight studies involved 1861 patients with HIV/AIDS-positive NTM isolates, including 23 cross-sectional studies, three case-control studies, one cohort study, and one case series. They were published between 2008 and 2022, with 15 (53.6%) being published between 2018 and 2022. The studies were conducted in 13 regions (provinces, municipalities directly under the central government, and autonomous regions), mostly in the southern region, chiefly Guangxi (**Figure 2**, **Table 1**, and Table S7 in the **Online Supplementary Document**). Most of the patients were men (87.3%; 95% CI = 83.2-91.0) (**Figure 3**).

**Table 1:** Essential information and quality assessment of included literature in systematic review of positive NTM from patients with HIV/AIDS in China

						Observed indicators								
**Study**	**Study design**	**Location (W/E/N/S/C)**	**Survey date**	**Total sample size**	**Men, n (%)**	**Clinical symptoms**		**Laboratory tests**		**Thoracic imaging manifestations**		**Species distribution**	**Treatment outcome**	
Song et al., 2011 [39]	Case-series study	Beijing (N)	2009-2010	5	4 (80)	No	Yes		Yes		Yes			No
Ding et al., 2022 [40]	Cross-sectional study	Beijing (N)	2016-2021	71	62 (87.3)	Yes	Yes		Yes		Yes			Yes
Wang et al., 2017 [41]	Cross-sectional study	Beijing (N)	2009-2015	33	28 (84.8)	No	Yes		Yes		No			Yes
Wu et al., 2017 [42]	Cross-sectional study	Guangdong (S)	2008-2015	31	28 (90.3)	Yes	Yes		Yes		Yes			No
Cao et al., 2021 [43]	Cross-sectional study	Guangdong (S)	2014-2019	43	38 (88.4)	Yes	Yes		No		Yes			No
Jiang et al., 2014 [44]	Cross-sectional study	Guangdong (S)	2006-2010	13	13 (100)	Yes	No		Yes		No			No
Meng et al., 2008 [45]	Cross-sectional study	Guangxi (S)	2006-2007	36	NA	Yes	Yes		Yes		No			Yes
Meng et al., 2018 [46]	Cross-sectional study	Guangxi (S)	2012-2015	29	19 (65.5)	Yes	Yes		Yes		No			Yes
R. Lan et al., 2011 [47]	Case-control study	Guangxi (S)	2005-2008	102	82 (80.4)	No	Yes		No		Yes			No
Zhang et al., 2011 [48]	Case-control study	Guangxi (S)	2006-2008	82	NA	Yes	No		No		No			No
Yin et al., 2015 [49]	Cross-sectional study	Guangxi (S)	2009-2012	97	77 (79.4)	Yes	No		Yes		No			Yes
Huang et al., 2022 [50]	Cross-sectional study	Guangxi (S)	2018-2019	11	NA	No	Yes		No		Yes			No
Zhou et al., 2013 [51]	Cross-sectional study	Guangxi (S)	2006-2010	135	106 (78.5)	No	No		No		Yes			No
Wang et al., 2022 [52]	Cross-sectional study	Hubei (C)	2019-2021	9	8 (88.9)	Yes	Yes		No		No			Yes
Li et al., 2016 [53]	Cross-sectional study	Hubei (C)	2012-2015	27	24 (88.9)	Yes	Yes		Yes		No			No
Deng et al., 2013 [54]	Cross-sectional study	Hunan (C)	2008-2011	63	NA	Yes	Yes		Yes		No			No
Wang et al., 2021 [55]	Cross-sectional study	Jiangsu (E)	2017-2020	97	NA	No	Yes		No		Yes			No
Huang et al., 2021 [56]	Cross-sectional study	Jiangxi (E)	2017-2020	22	16 (72.7)	No	Yes		Yes		Yes			No
Li, 2018 [57]	Cross-sectional study	Shaanxi (W)	2016-2017	50	50 (100)	Yes	Yes		Yes		No			No
Zhu et al., 2013 [58]	Cross-sectional study	Shanghai (E)	2007-2012	27	27 (100)	Yes	Yes		Yes		No			No
Sun et al., 2019 [59]	Cross-sectional study	Shanghai (E)	2006-2015	377	329 (87.3)	No	No		No		Yes			No
Tian at al., 2022 [60]	Case-control study	Shanghai (E)	2015-2021	169	161 (95.3)	No	Yes		No		No			No
Wang et al., 2019 [61]	Cross-sectional study	Sichuan (W)	2014-2018	59	50 (84.7)	Yes	Yes		Yes		Yes			No
Zhang et al., 2021 [62]	Cross-sectional study	Yunnan (W)	2012-2019	90	69 (76.7)	No	Yes		No		Yes			No
Li et al., 2018 [63]	Cross-sectional study	Chongqing (W)	2013-2015	23	16 (69.6)	Yes	Yes		Yes		No			No
Liu et al., 2021 [64]	Cross-sectional study	Chongqing (W)	2019-2020	44	34 (77.3)	Yes	Yes		No		Yes			No
Chou et al., 2011 [65]	Cross-sectional study	Taiwan (E)	2004-2008	22	21 (95.5)	Yes	No		No		Yes			No
Chiang et al., 2020 [66]	Retrospective cohort study	Taiwan (E)	1996-2016	94	86 (91.5)	Yes	No		Yes		No			No

NA – not available, W – western region, E – eastern region, N – northern region, S – southern region, C – central region

### Species distribution

The species distribution of NTM isolates positive was mainly *Mycobacterium avium* complex (MAC) (64.3%; 95% CI = 51.8-76.7), *Mycobacterium kansasii* (9.4%; 95% CI = 5.4-14.2), *Mycobacterium gordonae* (8.7%; 95% CI = 4.4-13.0), *Mycobacterium abscessus* complex (4.0%), and other mycobacterial species (16.0%) (**Table 2**).

**Table 2:** Results of a meta-analysis of species distribution among positive NTM isolates from patients with HIV/AIDS

			Heterogeneity		
**NTM species distribution**	**Pooled estimate (95% CI)**	**Number of studies**	***P*-value**	***I^2^***	**Event/total**
*Mycobacterium avium* complex	64.3 (51.8-76.7)	14	<0.001	95.0	522/834
*Mycobacterium abscessus* complex	4.0 (1.9-6.7)	10	0.049	46.9	29/645
*Mycobacterium kansasii*	9.4 (5.4-14.2)	11	<0.001	75.4	86/796
*Mycobacterium gordonae*	8.7 (4.4-13.0)	9	<0.001	79.3	68/666
Other NTM species*	16.0 (10.6-21.4)	13	<0.001	78.6	129/763

CI – confidence interval, NTM – nontuberculous mycobacterial

*All other NTM species accounted for less than the above four species.

### Clinical symptoms

The more common clinical symptoms included fever (68.5%; 95% CI = 61.8-75.1), cough or expectoration (67.0%; 95% CI = 54.5-79.5), appetite loss (49.4%; 95% CI = 1.7-97.1), weight loss (45.5%; 95% CI = 28.9-62.2), superficial lymphadenectasis (41.1%; 95% CI = 30.5-51.6), fatigue (38.2%; 95% CI = 18.1-58.3), dyspnoea (34.9%; 95% CI = 17.0-52.8), erythra (30.6%), abdominal pain or diarrhoea (27.4%), chest pain (24.3%), night sweats (17.4%), and haemoptysis (4.3%) (**Table 3**).

**Table 3:** Results of meta-analysis of clinical symptoms in positive NTM isolates from patients with HIV/AIDS

			Heterogeneity		
**Clinical symptoms**	**Pooled estimate (95% CI)**	**Number of studies**	***P*-value**	***I*^2^, %**	**Event/total, n/N**
Fever	68.5 (61.8-75.1)	16	<0.001	74.7	495/743
Cough or expectoration	67.0 (54.5-79.5)	14	<0.001	95.2	477/721
Dyspnoea	34.9 (17.0-52.8)	8	<0.001	95.2	163/433
Chest pain	24.3 (3.8-54.0)	3	<0.001	94.1	59/195
Abdominal pain or diarrhoea	27.4 (23.2-31.7)	7	0.164	34.6	118/415
Night sweats	17.4 (10.8-23.9)	6	0.032	59.1	59/342
Fatigue	38.2 (18.1-58.3)	10	<0.001	97.4	217/596
Erythra	30.6 (5.6-55.6)	3	<0.001	88.9	24/94
Weight loss	45.5 (28.9-62.2)	12	<0.001	96.1	280/623
Haemoptysis	4.3 (1.6-7.0)	4	0.845	0.0	10/215
Appetite loss	49.4 (1.7-97.1)	3	<0.001	98.5	62/166
Superficial lymphadenectasis	41.1 (30.5-51.6)	10	<0.001	78.7	143/381

CI – confidence interval

### Laboratory tests

In the laboratory tests, the haemoglobin count was 93.907 g/L (95% CI = 82.988-104.827 g/L) and CD4+ T cell count was 33.772 pieces/μL (95% CI = 15.289-52.255). Albumin (ALB) levels <35 g/L were observed in 55.6% (95% CI = 19.1-92.2) of studies, erythrocyte sedimentation rate (ESR)>20 mm/h in 91.4% (95% CI = 69.8-100.0), C-reactive protein (CRP)>6 mg/L in 82.5% (95% CI = 71.1-93.8), anaemia in 59.0% (95% CI = 38.1-79.8), CD4+ T cell count ≤50 pieces/μL in 70.3% (95% CI = 57.5-81.7), CD4+ T cell count 51-200 pieces/μL in 22.1% (95% CI = 14.8-30.3), and CD4+ T cell count >200 pieces/μL in 4.6% (95% CI = 0.8-10.3) (**Table 4** and **Table 5**).

**Table 4:** Results of meta-analysis of laboratory tests on positive NTM isolates from patients with HIV/AIDS

				Heterogeneity		
Laboratory tests*	**MD**	**95% CI**	**Number of studies**	***P*-value**	***I*^2^, %**	
Haemoglobin count (g/L)	93.907	82.988-104.827	5	<0.001		92.7
CD4^+^ T cell count (pieces/μL)	33.772	15.289-52.255	6	<0.001		99.3

CI – confidence interval, MD – mean deviation

*Continuous variables, mean deviation (95% CI).

**Table 5:** Results of meta-analysis of laboratory tests on positive NTM isolates from patients with HIV/AIDS

				Heterogeneity			
Laboratory tests*	**Pooled estimate (95% CI)**		**Number of studies**	***P*-value**	***I*^2^, %**		**Event/total**
ALB<35 (g/L)	55.6 (19.1-92.2)		4	<0.001		95.8	90/146
ESR>20 (mm/h)	91.4 (69.8-100.0)		3	0.001		84.7	117/135
CRP>6 (mg/L)	82.5 (71.1-93.8)		3	0.013		76.9	144/174
Anemia	59.0 (38.1-79.8)		7	<0.001		93.0	146/238
CD4^+^ T cell count ≤50 (pieces/μL)	70.3 (57.5-81.7)		13	<0.001		89.9	417/630
CD4+ cell count 51-200 (pieces/μL)	22.1 (14.8-30.3)		13	<0.001		78.6	153/630
CD4+ cell count >200 (pieces/μL)	4.6 (0.8-10.3)		13	<0.001		82.2	60/630

MD – mean deviation, CI – confidence interval, ALB – albumin, ESR – erythrocyte sedimentation rate, CRP – C-reactive protein

*Dichotomous variables, prevalence rate (95% CI).

### Thoracic imaging manifestations

Thoracic imaging manifestations (**Table 6**) show that the distribution of lesions was mainly bilateral lung involvement (83.8%; 95% CI = 70.7-93.9), followed by single lung involvement (12.8%; 95% CI = 5.1-22.8) and with no rare abnormalities (8.7%; 95% CI = 0.0-26.0). Furthermore, changes in lesion morphology and density mostly manifested as stripe shadow (60.3%; 95% CI = 41.9-77.4), patchy shadows (42.9%; 95% CI = 26.8-58.9), nodules (40.6%; 95% CI = 27.7-53.5), bronchiectasis (38.6%; 95% CI = 27.7-49.5), ground glass opacity (33.4%; 95% CI = 15.8-51.0), and some as cavitary lesions (13.0%), while millet shadows (4.6%) were rare. The accompanying signs were thoracic lymph node enlargement (49.5%; 95% CI = 25.8-73.3), abdominal lymph node enlargement (26.4%, 95% CI = 9.1-43.7), pleural thickening (14.9%; 95% CI = 8.1-21.7), hydrothorax (12.2%), and hydropericardium (11.2%).

**Table 6:** Results of meta-analysis of thoracic imaging manifestations in positive NTM isolates from patients with HIV/AIDS

			Heterogeneity		
**Thoracic imaging manifestations**	**Pooled estimate (95%CI)**	**Number of studies**	***P*-value**	***I*^2^, %**	**Event/total**
Distribution of lesions					
Single lung involvement	12.8 (5.1-22.8)	7	<0.001	75.2	45/297
Bilateral lung involvement	83.8 (70.7-93.9)	7	<0.001	83.4	240/297
No abnormalities	8.7 (0.0-26.0)	10	<0.001	95.3	85/446
Changes in lesion morphology and density					
*Patchy shadows*	42.9 (26.8-58.9)	8	<0.001	91.8	155/371
*Nodules*	40.6 (27.7-53.5)	10	<0.001	89.6	157/470
*Millet shadow*	4.6 (0.4-11.9)	8	<0.001	83.5	23/387
*Cavitary lesion*	13.0 (5.0-23.4)	14	<0.001	89.5	110/602
*Stripe shadow*	60.3 (41.9-77.4)	6	<0.001	88.7	144/291
*Ground glass opacity*	33.4 (15.8-51.0)	4	0.001	81.9	46/161
*Bronchiectasis*	38.6 (27.7-49.5)	6	0.034	58.6	84/219
Accompanying signs					
*Thoracic lymph node enlargement*	49.5 (25.8-73.3)	9	<0.001	94.5	148/346
*Abdominal lymph node enlargement*	26.4 (9.1-43.7)	3	0.002	84.2	45/150
*Hydropericardium*	11.2 (7.0-15.4)	5	0.357	8.7	27/213
*Hydrothorax*	12.2 (4.7-22.2)	10	<0.001	86.1	59/441
*Pleural thickening*	14.9 (8.1-21.7)	7	0.022	59.4	48/290

CI – confidence interval

### Treatment outcome

Analysis of treatment outcome showed that symptoms improved (70.0%; 95% CI = 56.9-83.0) in most patients after treatment, with death and other outcomes accounting for 6.2% (95% CI = 3.2-9.9) and 22.6% of total outcomes (**Table 7**).

**Table 7:** Results of meta-analysis of treatment outcome with positive NTM isolates from patients with HIV/AIDS

			Heterogeneity		
**Treatment outcome**	**Pooled estimate (95% CI)**	**Number of studies**	***P*-value**	***I*^2^, %**	**Event/total**
Symptoms improve*	70.0 (56.9-83.0)	6	<0.001	83.2	184/275
Death	6.2 (3.2-9.9)	6	0.377	6.2	20/275
Others†	22.6 (9.9-35.3)	6	<0.001	86.8	71/275

CI – confidence interval

*Symptom improvement is defined as getting better after treatment during hospitalisation.

†Others include automatic discharge, transfer to another hospital, and no apparent improvement.

### Subgroup analyses

We found differences between regions and some disease characteristics in the subgroup analyses (Table S8 and S9 in the **Online Supplementary Document**). For example, cough or expectoration was more frequent in the western (88.4%; 95% CI = 79.4-97.4) than the northern region (33.8%; 95% CI = 23.9-45.4, *P* < 0.001). We found no significant association between sample size (per study) and disease characteristics; however, fever (74.6%; 95% CI = 68.3-80.8 vs 61.6%; 95% CI = 50.6-72.7 (*P* = 0.045)), night sweats (33.2%; 95% CI =  17.1-49.3 vs 14.8%; 95% CI = 9.2-20.4 (*P* = 0.034)), and manifestations of stripe shadows on thoracic imaging (78.3%; 95% CI = 49.9-97.5 vs 44.0%; 95% CI = 27.6-61.1 (*P* = 0.042)) were more common in sample sizes of with <50 than those with ≥50 patients, while anaemia was less frequent in sample sizes with <50 than in ones with ≥50 patients (53.9%; 95% CI = 32.8-75.0 vs 86.4%; 95% CI = 75.5-93.0 (*P* = 0.005)).

Both subgroup analyses showed a high degree of heterogeneity and an *I*^2^≥50%. We hypothesise that the above-mentioned differences were caused by confounding factors outside the selected subgroup criteria, including data collection era, clinical typing (pulmonary NTM disease, extrapulmonary NTM disease, or disseminated NTM disease) and infection strain species.

## DISCUSSION

### Clinical significance of positive NTM isolates

Positive NTM isolation from clinical specimens at different sites has different implications: NTM isolated from non-sterile sites, such as sputum and bronchial lavage fluid, should exclude the possibility of specimen contamination or respiratory colonisation, while that from sterile sites, such as blood, cerebrospinal fluid, and puncture fluid, is more likely to be infectious or pathogenic [67,68]. When contamination of the specimen is excluded, positive NTM isolates include NTM colonisation, NTM infections, and NTM disease. As the immune status of the body changes, NTM colonisation or infections may progress to NTM disease, resulting in systemic tissue and organ damage.

### Initial screening strategies for NTM infections from patients with HIV/AIDS in China

Both TB infections and NTM infections can cause the same clinical symptoms, with the four clinical symptoms recommended by the World Health Organization for screening for TB infections including cough, fever, night sweats, and weight loss [69]. Moreover, a recent systematic review of patients co-infected with HIV and TB shows that C-reactive protein testing and chest imaging can be useful for screening TB infections [70]. There is no recommended screening strategy for NTM infections from patients with HIV/AIDS in China. We found that fever, cough or expectoration, appetite loss, weight loss, and superficial lymphadenectasis were the five most common clinical symptoms, while the incidence of haemoptysis was 4.3%, unlike TB, which is the leading cause of haemoptysis worldwide [71]. Therefore, these clinical symptoms can be used for the initial screening of NTM infections.

In our study, 55.6% of patients had an ALB<35 g/L, 91.4% had an ESR>20 mm/h, and 59% had anaemia, which was predominantly mild. Moreover, most patients had thoracic imaging involvement, and only 8.7% of patients had no thoracic imaging changes, mainly showing stripe shadows, patchy shadows, nodules, bronchiectasis, and signs of thoracic lymph node enlargement. However, some studies have found that acute infections of HIV/AIDS combined with MTB, with the thoracic imaging manifestations being mainly pneumonia-like exudates or solid shadow and nodules are rare [72]. Thus, thoracic imaging (x-ray or computed tomography) should be used as a routine and necessary means to diagnose NTM infections, regardless of whether they present with clinical symptoms, which is important for the early detection of the disease.

In summary, we consider that clinical symptoms, including fever, cough or expectoration, appetite loss, weight loss, and superficial lymphadenectasis without haemoptysis, laboratory tests, including ALB, erythrocyte sedimentation rate, and haemoglobin, and thoracic imaging are helpful in the initial screening for NTM infections.

### Priority population for screening for NTM infections from patients with HIV/AIDS in China

We found the CD4+ T cell count to be 33.772 pieces/μL (95% CI = 15.289-52.255 pieces/μL) in the included studies, with 70.3% of patients having a CD4+ T cell count ≤50 pieces/μL (95% CI = 57.5-81.7). Men comprised 87.3% of the total population. Water and soil are important transmission routes for NTM infections [24,25], and some species such as *Mycobacterium abscessus* can be transmitted interpersonally [73]. Therefore, we propose that patients with HIV/AIDS who are severely immunosuppressed (CD4+ T cell count <50 pieces/μL), especially men and patients with HIV/AIDS who are chronically exposed to unclean water sources in occupations related to exposure to soil (eg, farmers, gardeners) and have been or are being exposed to repeated NTM infections or NTM disease may be a priority population for screening for NTM infections.

### Principles of treatment for NTM disease from patients with HIV/AIDS in China

There is a lack of studies on the distribution of the NTM species over large areas of China. We found that the distribution of NTM species in China was dominated by *Mycobacterium avium* complex, which accounted for 64.3% of the species, while being predominant in different regions. *Mycobacterium avium* complex is a slow-growing mycobacterium. Therefore, when the results of the NTM species identification are unclear, experimental treatment for *Mycobacterium avium* complex infections may be feasible for critically ill patients or in cases when the disease is progressing rapidly. Physicians should also raise awareness of the diagnosis and treatment of *Mycobacterium avium* complex.

### Prophylactic treatment of NTM disease for patients with HIV/AIDS in China

Current Chinese expert consensus recommends prophylactic treatment for patients with HIV/AIDS with a CD4+ T cell count <50 pieces/μL [30], mainly based on guidelines and literature from high-income countries in Europe and North America that do not correspond to the circumstances in LMICs with scarce medical resources and weak economies. We found that approximately 30.0% of hospitalised patients failed to improve after treatment, making prophylactic treatment particularly important. Patients with HIV/AIDS should receive prophylactic anti-tuberculosis treatment with isoniazid, rifampicin, and rifapentine after active TB has been ruled out, regardless of the degree of immunosuppression or being tested for MTB infections [69]. Prophylactic treatment for NTM disease includes azithromycin, clarithromycin, and rifabutin [67,68]. We found that 22.1% of patients had a CD4+ T cell count of 51-200 pieces/μL, and the Food and Drug Administration showed weak drug interactions between clarithromycin, rifabutin, and the three prophylactic anti-tuberculosis drugs [74]. Therefore, it may be more appropriate to up-regulate CD4+ T cell count to ≤200 pieces/μL for the prophylactic treatment of NTM disease in patients with HIV/AIDS in China.

### Strengths and weakness

This is the first systematic review of positive NTM isolates from patients with HIV/AIDS in China. We followed PRISMA guidelines [31-33] in reporting and conducting the review, ensuring that all relevant studies are included. Consequently, it not only provides theoretical support for existing expert consensus in China, but also fills a relevant gap and provides information for future clinical research directions.

This study had certain limitations. Most of the included studies did not specify the type of clinical infection, and the selected control groups varied (eg, HIV/AIDS co-infection with NTM vs MTB and HIV/AIDS vs non-HIV/AIDS co-infection with NTM). Therefore, we were unable to make a definitive analysis of HIV/AIDS co-infection with NTM/MTB and HIV/AIDS co-infection with pulmonary NTM disease/extrapulmonary NTM disease/disseminated NTM disease. Additionally, the sample size of the included studies was too small, and the findings cannot be applied to all regions. Moreover, some of our recommendations are based on hypothetical reasoning inferred from our findings, and their specific clinical value and feasibility require further confirmation through cohort studies, clinical trials, and cost-benefit analyses.

## CONCLUSIONS

Focusing on clinical symptoms, laboratory tests, and thoracic imaging helps with initial screening for NTM infections. Physicians should raise awareness of the diagnosis and treatment of *Mycobacterium avium* complex, providing guidance for experimental treatment, screening of priority populations for NTM infections, and prophylactic treatment of NTM disease

## Additional material


Online Supplementary Document

## References

[1] GBD 2019 Diseases and Injuries Collaborators Global burden of 369 diseases and injuries in 204 countries and territories, 1990-2019: a systematic analysis for the Global Burden of Disease Study 2019. Lancet. 2020;396:1204-22. 10.1016/S0140-6736(20)30925-9 33069326 PMC7567026

[2] He Na [New progress in research on AIDS epidemiology in China]. Chinese Journal of Disease Control & Prevention. 2021;25:1365-8. Chinese.

[3] GBD 2017 HIV collaborators Global, regional, and national incidence, prevalence, and mortality of HIV, 1980-2017, and forecasts to 2030, for 195 countries and territories: a systematic analysis for the Global Burden of Diseases, Injuries, and Risk Factors Study 2017. Lancet HIV. 2019;6:e831-59. 10.1016/S2352-3018(19)30196-1 31439534 PMC6934077

[4] Yen YF Chen M Jen IA Chuang PH Lee CY Lin SI Short- and Long-term Risks of Highly Active Antiretroviral Treatment with Incident Opportunistic Infections among People Living with HIV/AIDS. Sci Rep. 2019;9:3476. 10.1038/s41598-019-39665-6 30837537 PMC6400900

[5] Buchacz K Lau B Jing Y Bosch R Abraham AG Gill MJ Incidence of AIDS-Defining Opportunistic Infections in a Multicohort Analysis of HIV-infected Persons in the United States and Canada, 2000-2010. J Infect Dis. 2016;214:862-72. 10.1093/infdis/jiw085 27559122 PMC4996145

[6] Hu J Gu L Shao Y Zhang R Qi T Sun J Long-term case-fatality rate of nontuberculous mycobacterial disease in people living with HIV. Infect Dis Poverty. 2022;11:16. 10.1186/s40249-022-00942-8 35130974 PMC8822711

[7] Echeverria G Rueda V Espinoza W Rosero C Zumárraga MJ de Waard JH First Case Reports of Nontuberculous Mycobacterial (NTM) Lung Disease in Ecuador: Important Lessons to Learn. Pathogens. 2023;12:507. 10.3390/pathogens12040507 37111393 PMC10142742

[8] Jing H Tan W Deng Y Gao D Li L Lu Z Diagnostic delay of pulmonary nontuberculous mycobacterial infection in China. Multidiscip Respir Med. 2014;9:48. 10.1186/2049-6958-9-48 25264489 PMC4177178

[9] Liu S Gao X Zhu J Chen J Yang H He L [Nontuberculous mycobacteria pulmonary disease: A retrospective analysis]. Zhong Nan Da Xue Xue Bao Yi Xue Ban. 2019;44:432-6. Chinese. 31113920 10.11817/j.issn.1672-7347.2019.04.014

[10] Bekker LG Alleyne G Baral S Cepeda J Daskalakis D Dowdy D Advancing global health and strengthening the HIV response in the era of the Sustainable Development Goals: the International AIDS Society-Lancet Commission. Lancet. 2018;392:312-58. 10.1016/S0140-6736(18)31070-5 30032975 PMC6323648

[11] B-Lajoie MR Drouin O Bartlett G Nguyen Q Low A Gavriilidis G Incidence and Prevalence of Opportunistic and Other Infections and the Impact of Antiretroviral Therapy Among HIV-infected Children in Low- and Middle-income Countries: A Systematic Review and Meta-analysis. Clin Infect Dis. 2016;62:1586-94. 10.1093/cid/ciw139 27001796 PMC4885647

[12] Okoi C Anderson ST Mulwa S Worwui A Antonio M Gehre F Pulmonary non-tuberculous mycobacteria in colonisation and disease in The Gambia. Sci Rep. 2022;12:19523. 10.1038/s41598-022-22777-x 36376401 PMC9663703

[13] Umrao J Singh D Zia A Saxena S Sarsaiya S Singh S Prevalence and species spectrum of both pulmonary and extrapulmonary nontuberculous mycobacteria isolates at a tertiary care center. Int J Mycobacteriol. 2016;5:288-93. 10.1016/j.ijmyco.2016.06.008 27847012

[14] National Technic Steering Group Of The Epidemiological Sampling Survey For Tuberculosis Duanmu H [Report on fourth national epidemiological sampling survey of tuberculosis]. Zhonghua Jie He He Hu Xi Za Zhi. 2002;25:3-7. Chinese. 11953089

[15] Technical Guidance Group of the Fifth National TB Epidemiological Survey, The Office of the Fifth National TB Epidemiological Survey [The fifth national tuberculosis epidemiological survey in 2010]. Chinese Journal of Antituberculosis. 2012;34:485-508. Chinese.

[16] Liu L Zhang R Tang Y Qi T Song W Wang Z The importance of non-tuberculous mycobacteria identification in Chinese patients infected with HIV. Biosci Trends. 2018;12:515-6. 10.5582/bst.2018.01254 30473561

[17] Hoza AS Mfinanga SG Rodloff AC Moser I König B Increased isolation of nontuberculous mycobacteria among TB suspects in Northeastern, Tanzania: public health and diagnostic implications for control programmes. BMC Res Notes. 2016;9:109. 10.1186/s13104-016-1928-3 26887928 PMC4756402

[18] Alemayehu A Kebede A Neway S Tesfaye E Zerihun B Getu M A glimpse into the genotype and clinical importance of non tuberculous mycobacteria among pulmonary tuberculosis patients: The case of Ethiopia. PLoS One. 2022;17:e0275159. 10.1371/journal.pone.0275159 36155559 PMC9512186

[19] Karamat A Ambreen A Ishtiaq A Tahseen S Rahman MA Mustafa T Isolation of non-tuberculous mycobacteria among tuberculosis patients, a study from a tertiary care hospital in Lahore, Pakistan. BMC Infect Dis. 2021;21:381. 10.1186/s12879-021-06086-8 33894767 PMC8070300

[20] Luo B Sun J Cai R Shen Y Liu L Wang J Spectrum of Opportunistic Infections and Risk Factors for In-Hospital Mortality of Admitted AIDS Patients in Shanghai. Medicine (Baltimore). 2016;95:e3802. 10.1097/MD.0000000000003802 27227959 PMC4902383

[21] Xiao J Gao G Li Y Zhang W Tian Y Huang Y Spectrums of opportunistic infections and malignancies in HIV-infected patients in tertiary care hospital, China. PLoS One. 2013;8:e75915. 10.1371/journal.pone.0075915 24204583 PMC3808390

[22] Pang W Shang P Li Q Xu J Bi L Zhong J Prevalence of Opportunistic Infections and Causes of Death among Hospitalized HIV-Infected Patients in Sichuan, China. Tohoku J Exp Med. 2018;244:231-42. 10.1620/tjem.244.231 29563388

[23] Haworth CS Banks J Capstick T Fisher AJ Gorsuch T Laurenson IF British Thoracic Society guidelines for the management of non-tuberculous mycobacterial pulmonary disease (NTM-PD). Thorax. 2017;72:ii1-64. 10.1136/thoraxjnl-2017-210927 29054853

[24] Loret JF Dumoutier N Non-tuberculous mycobacteria in drinking water systems: A review of prevalence data and control means. Int J Hyg Environ Health. 2019;222:628-34. 10.1016/j.ijheh.2019.01.002 30670342

[25] Walsh CM Gebert MJ Delgado-Baquerizo M Maestre FT Fierer N A Global Survey of Mycobacterial Diversity in Soil. Appl Environ Microbiol. 2019;85:e01180-19. 10.1128/AEM.01180-19 31253672 PMC6696970

[26] Martínez González S Cano Cortés A Sota Yoldi LA García García JM Alba Álvarez LM Palacios Gutiérrez JJ Non-Tuberculous Mycobacteria. An Emerging Threat? Arch Bronconeumol. 2017;53:554-60. 10.1016/j.arbr.2017.08.004 28433210

[27] Swenson C Zerbe CS Fennelly K Host Variability in NTM Disease: Implications for Research Needs. Front Microbiol. 2018;9:2901. 10.3389/fmicb.2018.02901 30559727 PMC6286975

[28] Wu ML Aziz DB Dartois V Dick T NTM drug discovery: status, gaps and the way forward. Drug Discov Today. 2018;23:1502-19. 10.1016/j.drudis.2018.04.001 29635026 PMC6078814

[29] Daley CL Iaccarino JM Lange C Cambau E Wallace RJ Jr Andrejak C Treatment of nontuberculous mycobacterial pulmonary disease: an official ATS/ERS/ESCMID/IDSA clinical practice guideline. Eur Respir J. 2020;56:2000535. 10.1183/13993003.00535-2020 32636299 PMC8375621

[30] AIDS Professional Group, Society of Tropical Disease and Parasitology of Chinese Medical Association [Expert consensus on diagnosis and treatment of HIV/AIDS patients combined with nontuberculous mycobacteria infection]. Chinese Journal of Infectious Diseases. 2019;37:129-38. Chinese.

[31] Knobloch K Yoon U Vogt PM Preferred reporting items for systematic reviews and meta-analyses (PRISMA) statement and publication bias. J Craniomaxillofac Surg. 2011;39:91-2. 10.1016/j.jcms.2010.11.001 21145753

[32] Page MJ McKenzie JE Bossuyt PM Boutron I Hoffmann TC Mulrow CD The PRISMA 2020 statement: an updated guideline for reporting systematic reviews. BMJ. 2021;372:n71. 10.1136/bmj.n71 33782057 PMC8005924

[33] Page MJ Moher D Bossuyt PM Boutron I Hoffmann TC Mulrow CD PRISMA 2020 explanation and elaboration: updated guidance and exemplars for reporting systematic reviews. BMJ. 2021;372:n160. 10.1136/bmj.n160 33781993 PMC8005925

[34] Hernandez AV Roman YM White CM Developing Criteria and Associated Instructions for Consistent and Useful Quality Improvement Study Data Extraction for Health Systems. J Gen Intern Med. 2020;35:802-7. 10.1007/s11606-020-06098-1 32808207 PMC7652974

[35] Wells GA, Shae B, O’Connell D, Peterson J, Welch V, Losos M. The Newcastle-Ottawa Scale (NOS) for assessing the quality of nonrandomised studies in meta-analyses. 2000. Available: http://www.ohri.ca/programs/clinical_epidemiology/oxford.asp. Accessed: 09 February 2023.

[36] Ma LL Chen WS Yang ZH Huang D Weng H Zeng XT Methodological quality (risk of bias) assessment tools for primary and secondary medical studies: what are they and which is better? Mil Med Res 2020;7:7. 10.1186/s40779-020-00238-8 32111253 PMC7049186

[37] Liu M Chen WS Liu YX Liu YQ Yuan YD Meta-analysis of single rates with zero events. Chinese Journal of Evidence-Based Medicine. 2020;20:1226-33. Chinese.

[38] Luo DH Wan X Liu JM Tong TJ [How to estimate the sample mean and standard deviation from the sample size, median, extremes or quartiles?]. Chinese Journal of Evidence-Based Medicine. 2017;17:1350-6. Chinese.

[39] Song WY Zhao DW Zhang T Imaging findings of non-tuberculous mycobacteria infection in AIDS: report of 5 cases. Journal of Practical Radiology. 2011;27:501-4.

[40] Ding XR Liu JC Chen SH Kang YF Wang CH Lou JL [Analysis of clinical characteristics of bloodstream infection of mycobacteria in AIDS patients]. Chinese Journal of Antituberculosis. 2022;44:821-6. Chinese.

[41] Wang F Guo JJ Xiang P Gao GJ Yang D Han N [Clinical features of AIDS patients with nontuberculous mycobacteria infection]. Infectious Disease Information. 2017;30:331-4. Chinese.

[42] Wu Y Deng XZ Hu FY Chen WS Chen XJ Cai WP [Pathogenic spectrum, clinical features and drug resistance of pneumonia caused by nontuberculous mycobacteria in acquired immunodeficiency syndrome patients]. Chinese Journal of Infectious Diseases. 2017;35:142-5. Chinese.

[43] Cao ML Chen F Wang FX [Clinical characteristics and drug-resistance analysis of complicating nontuberculous mycobacteria disease in AIDS patients]. Chinese Journal of Clinical Infectious Diseases. 2021;14:434-8. Chinese.

[44] Jiang GH Zhu Y Ye ZB Li CH Zhang GQ Ma XF [Explore chest radiographic appearances of non-tuberculous mycobacterial pulmonary infection in patients with AIDS]. China Journal of Emergency Resuscitation and Disaster Medicine. 2014;2:116-9. Chinese.

[45] Meng ZH-H Zhang FJ Liu CX Yang RY Li HR Yu L [A clinic analysis of 133 cases HIV infection/AIDS combined with mycobacterial pulmonary disease on divarication bacilli]. Journal of Clinical Internal Medicine. 2008;25:478-80. Chinese.

[46] Meng ZH-H Zuo Y Wu JW Shen Y-ZH [Clinical characteristics and prognosis of AIDS patients with nontuberculous mycobacterial disease]. Infectious Disease Information. 2018;31:544-7.

[47] Lan R Yang C Lan L Ou J Qiao K Liu F Mycobacterium tuberculosis and non-tuberculous mycobacteria isolates from HIV-infected patients in Guangxi, China. Int J Tuberc Lung Dis. 2011;15:1669-75. 10.5588/ijtld.11.0036 22118176

[48] Zhang Y Yu L Zhao Y Huang SH-B Tang ZH-R Liu W [Nontuberculous mycobacteria colonization in respiratory track of patients with HIV infection in Guangxi]. Chinese Journal of Experimental and Clinical Infectious Diseases. 2011;5:8-13. Chinese.

[49] Yin CH-L Xie ZH-H Pei J Zhang J Ou J [Clinical analysis of 97 HIV/AIDS patients with nontuberculous mycobacterial lung disease]. Infectious Disease Information. 2015;28:112-4. Chinese.

[50] Huang J Xie K Lu X-CH Xie ZH-H Lan YQ Wei LB [Comparative analysis of HIV-infected and non-HIV-infected patients with nontuberculous mycobacteria]. J Trop Med. 2022;22:1109-12. Chinese.

[51] Zhou CH-M Lan R-SH Liao GF Huang MY Zhao JM Luo D [Analysis of mycobacterium infections in 291 patients with HIV/AIDS]. Guangxi Medical Journal. 2013;35:29-31. Chinese.

[52] Wang SH Zhang YX Mo P-ZH Liang K Xiong Y [A report of 9 cases of HIV-associated Mycobacterium avium-intracellulare complex infection]. Chinese Journal of AIDS & STD. 2022;28:1083-4. Chinese.

[53] Li X Huang Y Lyu GY Yu HH Song ZH-Q [Chest imaging features of acquired immunodeficiency syndrome patients complicated with Mycobacterium avium-intracellulare complex infection]. Zhongguo Yiyuan Ganranxue Zazhi. 2016;26:2242-4. Chinese.

[54] Deng XJ Zhang X Yang L Yang H Ou S Lu L [Epidemiological investigation and clinical characteristics of AIDS-infected with nontuberculous mycobacteria]. China Foreign Medical Treatment. 2013;32:53-4. Chinese.

[55] Wang JJ Shi XD Xiao YY Zhang M Sui J Huang J [Distribution and drug resistance analysis of mycobacterium infection in HIV/AIDS patients in Nanjing area]. Chinese Journal of Clinical Laboratory Science. 2021;39:148-50. Chinese.

[56] Huang J Zhou M [Clinical analysis of Analysis of nontuberculous mycobacteria Infections in 57 Patients with AIDS]. Practical Clinical Medicine. 2021;22:18-9. Chinese.

[57] Li JY [Image features of chest CT in AIDS combined with non-tuberculous mycobacteria lung disease and tubercle bacillus]. Clinical Research and Practice. 2018;3:146-7. Chinese.

[58] Zhu Y Zhang ZH-Y Shi YX Feng F [CT features of pulmonary mycobacterial disease in patients with acquired immunodeficiency syndrome]. Zhonghua Fang She Xue Za Zhi. 2013;47:23-7.

[59] Sun JJ Le XQ Shen Y-ZH Zhang RF Wang JR Qi TK [Analysis of the positive results of mycobacterium culture detection among AIDS patients in Shanghai Public Health Clinical Center from 2006 to 2015]. Journal of Clinical Internal Medicine. 2019;36:733-5. Chinese.

[60] Tian B Shen Y-ZH Bai JS Liu J Chen HY Sun JJ [Clinical Characteristics of Talaromyces Marneffei Disease and Disseminated Non-tuberculosis Mycobacterium Disease in AIDS Patients]. Journal of Kunming Medical University. 2022;43:140-4. Chinese.

[61] Wang DM Liao Y Li QF Zhu M Wu GH Xu YH Drug resistance and pathogenic spectrum of patients coinfected with nontuberculous mycobacteria and human-immunodeficiency virus in Chengdu, China. Chin Med J (Engl). 2019;132:1293-7. 10.1097/CM9.0000000000000235 30925547 PMC6629352

[62] Zhang GX Gao L Li ZH-L Zhang M Xie Q [Analysis of distribution and immunological characteristics of HIV co-infected with non-tuberculosis mycobacteria patients based on gene chip technology]. International Journal of Laboratory Medicine. 2021;42:1176-9. Chinese.

[63] Li QS Lu YQ Luo YD Liu M Chen YK [Difference of clinical characteristics and prognosis between HIV complicated with Mycobacterium tuberculosis infection and HIV complicated with non-tuberculous mycobacteria infection]. Chinese Journal of AIDS & STD. 2018;24:916-8. Chinese.

[64] Liu M Li MJ Yu Q He K Wu YS Yang HH [Bacteria distribution and drug resistance of co-infection patients of human immunodeficiency virus and nontuberculous mycobacteria in Chongqing]. Chinese Journal of Antituberculosis. 2021;43:501-5. Chinese.

[65] Chou CH Chen HY Chen CY Huang CT Lai CC Hsueh PR Clinical features and outcomes of disseminated infections caused by non-tuberculous mycobacteria in a university hospital in Taiwan, 2004-2008. Scand J Infect Dis. 2011;43:8-14. 10.3109/00365548.2010.519345 20849364

[66] Chiang CH Lee GH Chiang TH Tang PU Fang CT Disseminated Mycobacterium avium complex infection as a differential diagnosis of tuberculosis in HIV patients. Int J Tuberc Lung Dis. 2020;24:922-7. 10.5588/ijtld.19.0602 33156759

[67] Haworth CS Banks J Capstick T Fisher AJ Gorsuch T Laurenson IF British Thoracic Society guidelines for the management of non-tuberculous mycobacterial pulmonary disease (NTM-PD). Thorax. 2017;72:ii1-64. 10.1136/thoraxjnl-2017-210927 29054853

[68] Daley CL Iaccarino JM Lange C Cambau E Wallace RJ Jr Andrejak C Treatment of nontuberculous mycobacterial pulmonary disease: an official ATS/ERS/ESCMID/IDSA clinical practice guideline. Eur Respir J. 2020;56:2000535. 10.1183/13993003.00535-2020 32636299 PMC8375621

[69] World Health Organization. WHO consolidated guidelines on tuberculosis: tuberculosis preventive treatment: Module 1: prevention. Geneva: World Health Organization; 2020. 32186832

[70] Dhana A Hamada Y Kengne AP Kerkhoff AD Rangaka MX Kredo T Tuberculosis screening among ambulatory people living with HIV: a systematic review and individual participant data meta-analysis. Lancet Infect Dis. 2022;22:507-18. 10.1016/S1473-3099(21)00387-X 34800394 PMC8942858

[71] Davidson K Shojaee S Managing Massive Hemoptysis. Chest. 2020;157:77-88. 10.1016/j.chest.2019.07.012 31374211

[72] Kwan CK Ernst JD HIV and tuberculosis: a deadly human syndemic. Clin Microbiol Rev. 2011;24:351-76. 10.1128/CMR.00042-10 21482729 PMC3122491

[73] Bryant JM Grogono DM Rodriguez-Rincon D Everall I Brown KP Moreno P Emergence and spread of a human-transmissible multidrug-resistant nontuberculous mycobacterium. Science. 2016;354:751-7. 10.1126/science.aaf8156 27846606 PMC5142603

[74] U.S. Food and Drug Adminstration. Drugs@FDA: FDA-Approved Drugs. 2023. Available: https://www.accessdata.fda.gov/scripts/cder/daf/index.cfm. Accessed: 12 June 2023.

