# Supplementary material for: What do the clinical features of positive nontuberculous mycobacteria isolates from patients with HIV/AIDS in China reveal? A systematic review and meta-analysis
Source: J Glob Health. 2023 Sep 1;13:04093. doi: 10.7189/jogh.13.04093 (PMC10472018; doi:10.7189/jogh.13.04093)
Supplement: Online Supplementary Document [file jogh-13-04093-s001.pdf]

**Supplementary materials for “What do the Clinical Features of Positive Nontuberculous Mycobacteria Isolates from Patients with HIV/AIDS in China reveal? A Sytstematic Review and meta-analysis”.**

**Table S1. All original data**

**Table S2. Database search strategies**

**Table S3. The Agency for Healthcare Research and Quality (AHRQ) Methodology Checklist for Cross-Sectional Study**

**Table S4. The Newcastle-Ottawa Scale (NOS) for cohort study**

**Table S5. The Newcastle-Ottawa Scale (NOS) for case-control study**

**Table S6. The Joanna Briggs Institute (JBI) Critical Appraisal Checklist for case series/case reports**

**Table S7. Quality assessment of the included studies**

**Table S8. Subgroup analyses of positive NTM isolates from patients with HIV/AIDS in different region**

**Table S9. Subgroup analyses of positive NTM isolates from patients with HIV/AIDS in sample size per study**

## Table S1. All original data

| Gender distribution | Study | Event | Total |
|---|---|---|---|
| | Song et al., 2011 | 4 | 5 |
| | Ding et al., 2022 | 62 | 71 |
| | Wang et al., 2017 | 28 | 33 |
| | Wu et al., 2017 | 28 | 31 |
| | Cao et al., 2021 | 38 | 43 |
| | Jiang et al., 2014 | 13 | 13 |
| | Meng et al., 2018 | 19 | 29 |
| | R. Lan et al., 2011 | 82 | 102 |
| | Yin et al., 2015 | 77 | 97 |
| | Zhou et al., 2013 | 106 | 135 |
| | Wang et al., 2022 | 8 | 9 |
| | Li et al., 2016 | 24 | 27 |
| | Huang et al., 2021 | 16 | 22 |
| | Li, 2018 | 50 | 50 |
| | Zhu et al., 2013 | 27 | 27 |
| | Sun et al., 2019 | 329 | 377 |
| | Tian at al., 2022 | 161 | 169 |
| | Wang et al., 2019 | 50 | 59 |
| | Zhang et al., 2021 | 69 | 90 |
| | Li et al., 2018 | 16 | 23 |
| | Liu et al., 2021 | 34 | 44 |
| | Chou et al., 2011 | 21 | 22 |
| | Chiang et al., 2020 | 86 | 94 |
| **Species distribution** | | | |
| ***Mycobacterium avium* complex** | Study | Event | Total |
| | Song et al., 2011 | 4 | 5 |
| | Ding et al., 2022 | 68 | 71 |
| | Wu et al., 2017 | 15 | 31 |
| | Cao et al., 2021 | 24 | 43 |
| | R. Lan et al., 2011 | 55 | 102 |
| | Huang et al., 2022 | 7 | 11 |
| | Zhou et al., 2013 | 79 | 135 |
| | Wang et al., 2021 | 78 | 97 |
| | Huang et al., 2021 | 10 | 22 |
| | Sun et al., 2019 | 33 | 102 |
| | Wang et al., 2019 | 31 | 59 |
| | Zhang et al., 2021 | 73 | 90 |
| | Liu et al., 2021 | 24 | 44 |
| | Chou et al., 2011 | 21 | 22 |
| ***Mycobacterium abscessus* complex** | Study | Event | Total |
| | Wu et al., 2017 | 2 | 31 |

| | | | |
|---|---|---|---|
| | Cao et al., 2021 | 1 | 43 |
| | R. Lan et al., 2011 | 3 | 102 |
| | Zhou et al., 2013 | 4 | 135 |
| | Wang et al., 2021 | 1 | 97 |
| | Huang et al., 2021 | 3 | 22 |
| | Wang et al., 2019 | 8 | 59 |
| | Zhang et al., 2021 | 4 | 90 |
| | Liu et al., 2021 | 3 | 44 |
| | Chou et al., 2011 | 0 | 22 |
| ***Mycobacterium kansasii*** | Study | Event | Total |
| | Ding et al., 2022 | 3 | 71 |
| | Wu et al., 2017 | 4 | 31 |
| | Cao et al., 2021 | 2 | 43 |
| | R. Lan et al., 2011 | 8 | 102 |
| | Zhou et al., 2013 | 10 | 135 |
| | Wang et al., 2021 | 7 | 97 |
| | Sun et al., 2019 | 25 | 102 |
| | Wang et al., 2019 | 16 | 59 |
| | Zhang et al., 2021 | 6 | 90 |
| | Liu et al., 2021 | 5 | 44 |
| | Chou et al., 2011 | 0 | 22 |
| ***Mycobacterium gordonae*** | Study | Event | Total |
| | Wu et al., 2017 | 1 | 31 |
| | Cao et al., 2021 | 3 | 43 |
| | R. Lan et al., 2011 | 9 | 102 |
| | Zhou et al., 2013 | 10 | 135 |
| | Wang et al., 2021 | 5 | 97 |
| | Huang et al., 2021 | 6 | 22 |
| | Sun et al., 2019 | 29 | 102 |
| | Zhang et al., 2021 | 2 | 90 |
| | Liu et al., 2021 | 3 | 44 |
| **Other NTM species*** | Study | Event | Total |
| | Song et al., 2011 | 1 | 5 |
| | Wu et al., 2017 | 9 | 31 |
| | Cao et al., 2021 | 13 | 43 |
| | R. Lan et al., 2011 | 27 | 102 |
| | Huang et al., 2022 | 4 | 11 |
| | Zhou et al., 2013 | 32 | 135 |
| | Wang et al., 2021 | 6 | 97 |
| | Huang et al., 2021 | 3 | 22 |
| | Sun et al., 2019 | 15 | 102 |
| | Wang et al., 2019 | 4 | 59 |
| | Zhang et al., 2021 | 5 | 90 |

| | Study | Event | Total |
|---|---|---|---|
| | Liu et al., 2021 | 9 | 44 |
| | Chou et al., 2011 | 1 | 22 |
| **Clinical symptoms** | | | |
| **Fever** | Study | Event | Total |
| | Ding et al., 2022 | 54 | 71 |
| | Wu et al., 2017 | 25 | 31 |
| | Cao et al., 2021 | 34 | 43 |
| | Jiang et al., 2014 | 10 | 13 |
| | Meng et al., 2008 | 26 | 36 |
| | Meng et al., 2018 | 24 | 29 |
| | Zhang et al., 2011 | 42 | 82 |
| | Yin et al., 2015 | 70 | 97 |
| | Wang et al., 2022 | 5 | 9 |
| | Li et al., 2016 | 21 | 27 |
| | Deng et al., 2013 | 24 | 63 |
| | Wang et al., 2019 | 36 | 59 |
| | Li et al., 2018 | 19 | 23 |
| | Liu et al., 2021 | 30 | 44 |
| | Chou et al., 2011 | 10 | 22 |
| | Chiang et al., 2020 | 65 | 94 |
| **Cough or expectoration** | Study | Event | Total |
| | Ding et al., 2022 | 24 | 71 |
| | Wu et al., 2017 | 25 | 31 |
| | Cao et al., 2021 | 31 | 43 |
| | Meng et al., 2008 | 15 | 36 |
| | Meng et al., 2018 | 19 | 29 |
| | Zhang et al., 2011 | 52 | 82 |
| | Yin et al., 2015 | 93 | 97 |
| | Li et al., 2016 | 22 | 27 |
| | Deng et al., 2013 | 21 | 63 |
| | Wang et al., 2019 | 47 | 59 |
| | Li et al., 2018 | 22 | 23 |
| | Liu et al., 2021 | 39 | 44 |
| | Chou et al., 2011 | 8 | 22 |
| | Chiang et al., 2020 | 59 | 94 |
| **Dyspnea** | Study | Event | Total |
| | Ding et al., 2022 | 9 | 71 |
| | Cao et al., 2021 | 18 | 43 |
| | Meng et al., 2018 | 6 | 29 |
| | Yin et al., 2015 | 71 | 97 |
| | Li et al., 2016 | 10 | 27 |
| | Deng et al., 2013 | 7 | 63 |
| | Wang et al., 2019 | 20 | 59 |

| | | | |
|---|---|---|---|
| | Liu et al., 2021 | 22 | 44 |
| **Chest pain** | Study | Event | Total |
| | Ding et al., 2022 | 7 | 71 |
| | Yin et al., 2015 | 47 | 97 |
| | Li et al., 2016 | 5 | 27 |
| **Abdominal pain or diarrhea** | Study | Event | Total |
| | Ding et al., 2022 | 18 | 71 |
| | Cao et al., 2021 | 14 | 43 |
| | Meng et al., 2018 | 8 | 29 |
| | Yin et al., 2015 | 36 | 97 |
| | Wang et al., 2019 | 10 | 59 |
| | Chou et al., 2011 | 6 | 22 |
| | Chiang et al., 2020 | 26 | 94 |
| **Night sweats** | Study | Event | Total |
| | Ding et al., 2022 | 6 | 71 |
| | Zhang et al., 2011 | 16 | 82 |
| | Wang et al., 2022 | 2 | 9 |
| | Deng et al., 2013 | 13 | 63 |
| | Li et al., 2018 | 9 | 23 |
| | Chiang et al., 2020 | 13 | 94 |
| **Fatigue** | Study | Event | Total |
| | Ding et al., 2022 | 23 | 71 |
| | Wu et al., 2017 | 8 | 31 |
| | Cao et al., 2021 | 12 | 43 |
| | Meng et al., 2018 | 28 | 29 |
| | Zhang et al., 2011 | 31 | 82 |
| | Yin et al., 2015 | 58 | 97 |
| | Li et al., 2016 | 9 | 27 |
| | Deng et al., 2013 | 20 | 63 |
| | Wang et al., 2019 | 9 | 59 |
| | Chiang et al., 2020 | 19 | 94 |
| **Erythra** | Study | Event | Total |
| | Jiang et al., 2014 | 9 | 13 |
| | Wang et al., 2019 | 13 | 59 |
| | Chou et al., 2011 | 2 | 22 |
| **Weight loss** | Study | Event | Total |
| | Ding et al., 2022 | 10 | 71 |
| | Wu et al., 2017 | 17 | 31 |
| | Cao et al., 2021 | 3 | 43 |
| | Meng et al., 2018 | 23 | 29 |
| | Zhang et al., 2011 | 45 | 82 |
| | Yin et al., 2015 | 69 | 97 |
| | Wang et al., 2022 | 3 | 9 |

| | | | | |
|---|---|---|---|---|
| | Deng et al., 2013 | 19 | 63 | |
| | Wang et al., 2019 | 17 | 59 | |
| | Li et al., 2018 | 21 | 23 | |
| | Chou et al., 2011 | 7 | 22 | |
| | Chiang et al., 2020 | 46 | 94 | |
| **Hemoptysis** | Study | Event | Total | |
| | 5Cao et al., 2021 | 3 | 43 | |
| | Zhang et al., 2011 | 4 | 82 | |
| | Li et al., 2016 | 1 | 27 | |
| | Deng et al., 2013 | 2 | 63 | |
| **Appetite loss** | Study | Event | Total | |
| | Cao et al., 2021 | 14 | 43 | |
| | Meng et al., 2018 | 27 | 29 | |
| | Chiang et al., 2020 | 21 | 94 | |
| **Superficial lymphadenectasis** | Study | Event | Total | |
| | Cao et al., 2021 | 8 | 43 | |
| | Jiang et al., 2014 | 6 | 13 | |
| | Meng et al., 2018 | 16 | 29 | |
| | Yin et al., 2015 | 39 | 97 | |
| | Wang et al., 2022 | 6 | 9 | |
| | Li et al., 2016 | 11 | 27 | |
| | Deng et al., 2013 | 12 | 63 | |
| | Li, 2018 | 18 | 50 | |
| | Zhu et al., 2013 | 11 | 27 | |
| | Li et al., 2018 | 16 | 23 | |
| **Laboratory tests** | | | | |
| **ALB< 35 (g/L)** [b] | Study | Event | Total | |
| | Ding et al., 2022 | 63 | 71 | |
| | Cao et al., 2021 | 12 | 43 | |
| | Wang et al., 2022 | 6 | 9 | |
| | Li et al., 2018 | 9 | 23 | |
| **ESR＞20 (mm/h)** [b] | Study | Event | Total | |
| | Song et al., 2011 | 5 | 5 | |
| | Ding et al., 2022 | 68 | 71 | |
| | Wang et al., 2019 | 44 | 59 | |
| **CRP＞6 (mg/L)** [b] | Study | Event | Total | |
| | Ding et al., 2022 | 63 | 71 | |
| | Wang et al., 2019 | 52 | 59 | |
| | Liu et al., 2021 | 29 | 44 | |
| **Hemoglobin count (g/L)**[a] | Study | Mean | SD | Total |
| | Ding et al., 2022 | 78.70 | 14.60 | 71 |
| | Wang et al., 2017 | 93.80 | 22.90 | 33 |
| | Huang et al., 2022 | 91.79 | 32.23 | 11 |

| | | | | |
|---|---|---|---|---|
| | Huang et al., 2021 | 124.45 | 31.20 | 22 |
| | Tian at al., 2022 | 87.11 | 28.04 | 169 |
| **CD4+ T cell count (pieces/μL)[a]** | Study | Mean | SD | Total |
| | Wang et al., 2017 | 112.32 | 230.16 | 33 |
| | Huang et al., 2022 | 26.46 | 34.78 | 11 |
| | Huang et al., 2021 | 13.25 | 7.48 | 22 |
| | Li, 2018 | 42.63 | 6.28 | 50 |
| | Tian at al., 2022 | 10.11 | 11.96 | 168 |
| | Li et al., 2018 | 72.70 | 72.90 | 23 |
| **Anemia[b]** | Study | Event | Total | |
| | Wu et al., 2017 | 21 | 31 | |
| | Cao et al., 2021 | 10 | 43 | |
| | Meng et al., 2018 | 9 | 29 | |
| | Wang et al., 2022 | 5 | 9 | |
| | Wang et al., 2019 | 51 | 59 | |
| | Li et al., 2018 | 16 | 23 | |
| | Liu et al., 2021 | 34 | 44 | |
| **CD4+ T cell count ≤ 50 (pieces/μL)[b]** | Study | Event | Total | |
| | Song et al., 2011 | 2 | 5 | |
| | Ding et al., 2022 | 62 | 71 | |
| | Cao et al., 2021 | 31 | 43 | |
| | Meng et al., 2008 | 31 | 36 | |
| | Meng et al., 2018 | 23 | 29 | |
| | R. Lan et al., 2011 | 53 | 74 | |
| | Wang et al., 2022 | 3 | 9 | |
| | Li et al., 2016 | 19 | 27 | |
| | Deng et al., 2013 | 23 | 63 | |
| | Wang et al., 2021 | 51 | 97 | |
| | Zhu et al., 2013 | 27 | 27 | |
| | Wang et al., 2019 | 50 | 59 | |
| | Zhang et al., 2021 | 42 | 90 | |
| **CD4+ cell count 51–200 (pieces/μL)[b]** | Study | Event | Total | |
| | Song et al., 2011 | 3 | 5 | |
| | Ding et al., 2022 | 8 | 71 | |
| | Cao et al., 2021 | 10 | 43 | |
| | Meng et al., 2008 | 4 | 36 | |
| | Meng et al., 2018 | 6 | 29 | |
| | R. Lan et al., 2011 | 15 | 74 | |
| | Wang et al., 2022 | 5 | 9 | |
| | Li et al., 2016 | 8 | 27 | |
| | Deng et al., 2013 | 22 | 63 | |
| | Wang et al., 2021 | 27 | 97 | |
| | Zhu et al., 2013 | 0 | 27 | |

| | | | |
|---|---|---|---|
| | Wang et al., 2019 | 9 | 59 |
| | Zhang et al., 2021 | 36 | 90 |
| **CD4+ cell count ＞200 (pieces/μL)[b]** | Study | Event | Total |
| | Song et al., 2011 | 0 | 5 |
| | Ding et al., 2022 | 1 | 71 |
| | Cao et al., 2021 | 2 | 43 |
| | Meng et al., 2008 | 1 | 36 |
| | Meng et al., 2018 | 0 | 29 |
| | R. Lan et al., 2011 | 6 | 74 |
| | Wang et al., 2022 | 1 | 9 |
| | Li et al., 2016 | 0 | 27 |
| | Deng et al., 2013 | 18 | 63 |
| | Wang et al., 2021 | 19 | 97 |
| | Zhu et al., 2013 | 0 | 27 |
| | Wang et al., 2019 | 0 | 59 |
| | Zhang et al., 2021 | 12 | 90 |
| **Thoracic imaging manifestations** | | | |
| **Distribution of lesions** | | | |
| **Single lung involvement** | Study | Event | Total |
| | Ding et al., 2022 | 9 | 71 |
| | Jiang et al., 2014 | 0 | 13 |
| | Meng et al., 2008 | 4 | 10 |
| | Meng et al., 2018 | 10 | 29 |
| | Yin et al., 2015 | 14 | 97 |
| | Li, 2018 | 8 | 50 |
| | Zhu et al., 2013 | 0 | 27 |
| **Bilateral lung involvement** | Study | Event | Total |
| | Ding et al., 2022 | 54 | 71 |
| | Jiang et al., 2014 | 13 | 13 |
| | Meng et al., 2008 | 6 | 10 |
| | Meng et al., 2018 | 15 | 29 |
| | Yin et al., 2015 | 83 | 97 |
| | Li, 2018 | 42 | 50 |
| | Zhu et al., 2013 | 27 | 27 |
| **No abnormalities** | Study | Event | Total |
| | Ding et al., 2022 | 8 | 71 |
| | Wang et al., 2017 | 9 | 33 |
| | Jiang et al., 2014 | 0 | 13 |
| | Meng et al., 2008 | 0 | 10 |
| | Meng et al., 2018 | 4 | 29 |
| | Yin et al., 2015 | 0 | 97 |
| | Huang et al., 2021 | 4 | 22 |
| | Li, 2018 | 0 | 50 |

| | | | |
|---|---|---|---|
| | Zhu et al., 2013 | 0 | 27 |
| | Chiang et al., 2020 | 60 | 94 |
| **Changes of lesion morphology and density** | | | |
| **Patchy shadows** | Study | Event | Total |
| | Ding et al., 2022 | 30 | 71 |
| | Wu et al., 2017 | 25 | 31 |
| | Jiang et al., 2014 | 4 | 13 |
| | Yin et al, 2015 | 35 | 97 |
| | Li, 2018 | 18 | 50 |
| | Zhu et al., 2013 | 2 | 27 |
| | Wang et al., 2019 | 25 | 59 |
| | Li et al., 2018 | 16 | 23 |
| **Nodules** | Study | Event | Total |
| | Song et al., 2011 | 3 | 5 |
| | Ding et al., 2022 | 26 | 71 |
| | Wu et al., 2017 | 11 | 31 |
| | Jiang et al., 2014 | 8 | 13 |
| | Yin et al., 2015 | 20 | 97 |
| | 21Li, 2018 | 32 | 50 |
| | Zhu et al., 2013 | 18 | 27 |
| | Wang et al., 2019 | 19 | 59 |
| | Li et al., 2018 | 9 | 23 |
| | Chiang et al., 2020 | 11 | 94 |
| **Millet shadow** | Study | Event | Total |
| | Ding et al., 2022 | 1 | 71 |
| | Wang et al., 2017 | 4 | 33 |
| | Wu et al., 2017 | 2 | 31 |
| | Huang et al., 2021 | 5 | 22 |
| | Li, 2018 | 0 | 50 |
| | Zhu et al., 2013 | 0 | 27 |
| | Wang et al., 2019 | 11 | 59 |
| | Chiang et al., 2020 | 0 | 94 |
| **Cavitary lesion** | Study | Event | Total |
| | Song et al., 2011 | 1 | 5 |
| | Ding et al., 2022 | 2 | 71 |
| | Wang et al., 2017 | 0 | 33 |
| | Wu et al., 2017 | 7 | 31 |
| | Jiang et al., 2014 | 2 | 13 |
| | Meng et al., 2008 | 2 | 36 |
| | Meng et al., 2018 | 5 | 29 |
| | Yin et al., 2015 | 53 | 97 |
| | Deng et al., 2013 | 12 | 35 |

| | | | |
|---|---|---|---|
| | Huang et al., 2021 | 0 | 22 |
| | Li, 2018 | 8 | 50 |
| | Zhu et al., 2013 | 4 | 27 |
| | Wang et al., 2019 | 7 | 59 |
| | Chiang et al., 2020 | 7 | 94 |
| **Stripe shadow** | Study | Event | Total |
| | Ding et al., 2022 | 21 | 71 |
| | Wang et al., 2017 | 21 | 33 |
| | Jiang et al., 2014 | 13 | 13 |
| | Yin et al., 2015 | 41 | 97 |
| | Li, 2018 | 31 | 50 |
| | Zhu et al., 2013 | 17 | 27 |
| **Ground glass opacity** | Study | Event | Total |
| | Ding et al., 2022 | 10 | 71 |
| | Jiang et al., 2014 | 7 | 13 |
| | Li, 2018 | 19 | 50 |
| | Zhu et al., 2013 | 10 | 27 |
| **Bronchiectasis** | Study | Event | Total |
| | Song et al., 2011 | 1 | 5 |
| | Jiang et al., 2014 | 4 | 13 |
| | Yin et al., 2015 | 35 | 97 |
| | Li et al., 2016 | 18 | 27 |
| | Li, 2018 | 17 | 50 |
| | Zhu et al., 2013 | 9 | 27 |
| **Accompanying Signs** | | | |
| **Thoracic lymph node enlargement** | Study | Event | Total |
| | Song et al., 2011 | 5 | 5 |
| | Ding et al., 2022 | 51 | 71 |
| | Wu et al., 2017 | 25 | 31 |
| | Jiang et al., 2014 | 4 | 13 |
| | Meng et al., 2018 | 5 | 29 |
| | Yin et al., 2015 | 7 | 97 |
| | Li, 2018 | 28 | 50 |
| | Zhu et al., 2013 | 8 | 27 |
| | Li et al., 2018 | 15 | 23 |
| **Abdominal lymph node enlargement** | Study | Event | Total |
| | Ding et al., 2022 | 31 | 71 |
| | Meng et al., 2018 | 5 | 29 |
| | Li, 2018 | 9 | 50 |
| **Hydropericardium** | Study | Event | Total |
| | Ding et al., 2022 | 10 | 71 |
| | Wu et al., 2017 | 2 | 31 |
| | Meng et al., 2018 | 3 | 29 |

| | | | |
|---|---|---|---|
| | Wang et al., 2019 | 6 | 59 |
| | Li et al., 2018 | 6 | 23 |
| **Hydrothorax** | Study | Event | Total |
| | Ding et al., 2022 | 15 | 71 |
| | Wu et al., 2017 | 9 | 31 |
| | Meng et al., 2018 | 2 | 29 |
| | Deng et al., 2013 | 4 | 35 |
| | Huang et al., 2021 | 0 | 22 |
| | Li, 2018 | 2 | 50 |
| | Zhu et al., 2013 | 1 | 27 |
| | Wang et al., 2019 | 10 | 59 |
| | Li et al., 2018 | 14 | 23 |
| | Chiang et al., 2020 | 2 | 94 |
| **Pleural thickening** | Study | Event | Total |
| | Song et al., 2011 | 1 | 5 |
| | Ding et al., 2022 | 20 | 71 |
| | Jiang et al., 2014 | 5 | 13 |
| | Yin et al., 2015 | 13 | 97 |
| | Li et al., 2016 | 3 | 27 |
| | Li, 2018 | 4 | 50 |
| | Zhu et al., 2013 | 2 | 27 |
| **Treatment outcome** | | | |
| **Symptoms improve†** | Study | Event | Total |
| | Ding et al., 2022 | 45 | 71 |
| | Wang et al., 2017 | 28 | 33 |
| | Meng et al., 2008 | 13 | 36 |
| | Meng et al., 2018 | 23 | 29 |
| | Yin et al., 2015 | 67 | 97 |
| | Wang et al., 2022 | 8 | 9 |
| **Death** | Study | Event | Total |
| | Ding et al., 2022 | 2 | 71 |
| | Wang et al., 2017 | 2 | 33 |
| | Meng et al., 2008 | 3 | 36 |
| | Meng et al., 2018 | 4 | 29 |
| | Yin et al., 2015 | 9 | 97 |
| | Wang et al., 2022 | 0 | 9 |
| **Others‡** | Study | Event | Total |
| | Ding et al., 2022 | 24 | 71 |
| | Wang et al., 2017 | 3 | 33 |
| | Meng et al., 2008 | 20 | 36 |
| | Meng et al., 2018 | 2 | 29 |
| | Yin et al., 2015 | 21 | 97 |
| | Wang et al., 2022 | 1 | 9 |

NTM – nontuberculous mycobacterial, ALB – albumin, ESR – erythrocyte sedimentation Rate, CRP – C-reactive protein, SD – standard deviation

* All other NTM species accounted for less than the above four species.

†Symptom improve is defined as getting better after treatment during hospitalization.

‡Others include automatic discharge, transfer to another hospital, and no apparent improvement.

**Table S2. Database search strategies***

| PubMed | Search strategies |
|---|---|
| #1 | "HIV"[Mesh] Sort by: Most Recent |
| #2 | ((((((((((((((((((((((((Human Immunodeficiency Virus[Title/Abstract]) OR (Immunodeficiency Virus, Human[Title/Abstract])) OR (Immunodeficiency Viruses, Human[Title/Abstract])) OR (Virus, Human Immunodeficiency[Title/Abstract])) OR (Viruses, Human Immunodeficiency[Title/Abstract])) OR (Human Immunodeficiency Viruses[Title/Abstract])) OR (Human T Cell Lymphotropic Virus Type III[Title/Abstract])) OR (Human T-Cell Lymphotropic Virus Type III[Title/Abstract])) OR (Human T-Cell Leukemia Virus Type III[Title/Abstract])) OR (Human T Cell Leukemia Virus Type III[Title/Abstract])) OR (LAV-HTLV-III[Title/Abstract])) OR (Lymphadenopathy-Associated Virus[Title/Abstract])) OR (Lymphadenopathy Associated Virus[Title/Abstract])) OR (Lymphadenopathy-Associated Viruses[Title/Abstract])) OR (Virus, Lymphadenopathy-Associated[Title/Abstract])) OR (Viruses, Lymphadenopathy-Associated[Title/Abstract])) OR (Human T Lymphotropic Virus Type III[Title/Abstract])) OR (Human T-Lymphotropic Virus Type III[Title/Abstract])) OR (AIDS Virus[Title/Abstract])) OR (AIDS Viruses[Title/Abstract])) OR (Virus, AIDS[Title/Abstract])) OR (Viruses, AIDS[Title/Abstract])) OR (Acquired Immune Deficiency Syndrome Virus[Title/Abstract])) OR (Acquired Immunodeficiency Syndrome Virus[Title/Abstract])) OR (HTLV-III [Title/Abstract]) |
| #3 | #1 OR #2 |
| #4 | "Acquired Immunodeficiency Syndrome"[Mesh] Sort by: Most Recent |
| #5 | ((((((((((((((Immunologic Deficiency Syndrome, Acquired[Title/Abstract]) OR (Acquired Immune Deficiency Syndrome[Title/Abstract])) OR (Acquired Immuno-Deficiency Syndrome[Title/Abstract])) OR (Acquired Immuno Deficiency Syndrome[Title/Abstract])) OR (Acquired Immuno-Deficiency Syndromes[Title/Abstract])) OR (Immuno-Deficiency Syndrome, Acquired[Title/Abstract])) OR (Immuno-Deficiency Syndromes, Acquired[Title/Abstract])) OR (Syndrome, Acquired Immuno-Deficiency[Title/Abstract])) OR (Syndromes, Acquired Immuno-Deficiency[Title/Abstract])) OR (Immunodeficiency Syndrome, Acquired[Title/Abstract])) OR (Acquired Immunodeficiency Syndromes[Title/Abstract])) OR (Immunodeficiency Syndromes, Acquired[Title/Abstract])) OR (Syndrome, Acquired Immunodeficiency[Title/Abstract])) OR (Syndromes, Acquired Immunodeficiency[Title/Abstract])) OR (AIDS[Title/Abstract]) |
| #6 | #4 OR #5 |
| #7 | "Nontuberculous Mycobacteria"[Mesh] Sort by: Most Recent |
| #8 | ((((((((((((((((((Mycobacterium, Atypical[Title/Abstract]) OR (Tuberculoid Bacillus[Title/Abstract])) OR (Atypical Mycobacterium[Title/Abstract])) OR (Atypical Mycobacteria[Title/Abstract])) OR (Mycobacteria, Atypical[Title/Abstract])) OR (Nontuberculous Mycobacterium[Title/Abstract])) OR (Non-Tuberculous Mycobacteria[Title/Abstract])) OR (Mycobacterium szulgai[Title/Abstract])) OR (Mycobacterium gordonae[Title/Abstract])) OR (Mycobacterium duvalii[Title/Abstract])) OR (Mycolicibacterium duvalii[Title/Abstract])) OR (Mycobacterium flavescens[Title/Abstract])) OR (Mycolicibacterium flavescens[Title/Abstract])) OR (Mycobacterium gilvum[Title/Abstract])) OR (Mycolicibacterium gilvum[Title/Abstract])) OR (Mycobacterium obuense[Title/Abstract])) OR (Mycolicibacterium obuense[Title/Abstract])) OR (Mycobacterium terrae[Title/Abstract])) OR (Mycolicibacter terrae[Title/Abstract]) |
| #9 | #7 OR #8 |
| #10 | "China"[Mesh] Sort by: Most Recent |
| #11 | "Hong Kong"[Mesh] Sort by: Most Recent |
| #12 | "Macau"[Mesh] Sort by: Most Recent |
| #13 | "Taiwan"[Mesh] Sort by: Most Recent |

| | |
|---|---|
| #14 | #10 OR #11 OR #12 OR #13 |
| #15 | #3 OR #6 AND #9 AND #14 |
| **Embase** | |
| #1 | Emtree: 'human immunodeficiency virus'/exp |
| #2 | Title or Abstract: 'human immunodeficiency virus' OR 'immunodeficiency virus, human' OR 'immunodeficiency viruses, human' OR 'virus, human immunodeficiency' OR 'viruses, human immunodeficiency' OR 'human immunodeficiency viruses' OR 'human t cell lymphotropic virus type iii' OR 'human t-cell lymphotropic virus type iii' OR 'human t-cell leukemia virus type iii' OR 'human t cell leukemia virus type iii' OR 'lav htlv iii' OR 'lymphadenopathy-associated virus' OR 'lymphadenopathy associated virus' OR 'lymphadenopathy-associated viruses' OR 'virus, lymphadenopathy-associated' OR 'viruses, lymphadenopathy-associated' OR 'human t lymphotropic virus type iii' OR 'human t-lymphotropic virus type iii' OR 'aids virus' OR 'aids viruses' OR 'virus, aids' OR 'viruses, aids' OR 'acquired immune deficiency syndrome virus' OR 'acquired immunodeficiency syndrome virus' OR 'HTLV-III' |
| #3 | #1 OR #2 |
| #4 | Emtree: 'acquired immune deficiency syndrome'/exp |
| #5 | Title or Abstract: 'immunologic deficiency syndrome, acquired' OR 'acquired immune deficiency syndrome' OR 'acquired immuno-deficiency syndrome' OR 'acquired immuno deficiency syndrome' OR 'acquired immuno-deficiency syndromes' OR 'immuno-deficiency syndrome, acquired' OR 'immuno-deficiency syndromes, acquired' OR 'syndrome, acquired immuno-deficiency' OR 'syndromes, acquired immuno-deficiency' OR 'immunodeficiency syndrome, acquired' OR 'acquired immunodeficiency syndromes' OR 'immunodeficiency syndromes, acquired' OR 'syndrome, acquired immunodeficiency' OR 'syndromes, acquired immunodeficiency' OR aids |
| #6 | #4 OR #5 |
| #7 | Emtree: 'atypical mycobacteriosis'/exp |
| #8 | Title or Abstract: 'mycobacterium, atypical' OR 'tuberculoid bacillus' OR 'atypical mycobacterium' OR 'atypical mycobacteria' OR 'mycobacteria, atypical' OR 'nontuberculous mycobacterium' OR 'non-tuberculous mycobacteria' OR 'mycobacterium szulgai' OR 'mycobacterium gordonae' OR 'mycobacterium duvalii' OR 'mycolicibacterium duvalii' OR 'mycobacterium flavescens' OR 'mycolicibacterium flavescens' OR 'mycobacterium gilvum' OR 'mycolicibacterium gilvum' OR 'mycobacterium obuense' OR 'mycolicibacterium obuense' OR 'mycobacterium terrae' OR 'mycolicibacter terrae' |
| #9 | #7 OR #8 |
| #10 | Emtree: 'china'/exp |
| #11 | Emtree: 'hong kong'/exp |
| #12 | Emtree: 'macao'/exp |
| #13 | Emtree: 'taiwan'/exp |
| #14 | #10 OR #11 OR #12 OR #13 |
| #15 | #3 OR #6 |
| #16 | #9 AND #14 AND #15 |
| **Web of Science** | |
| #1 | Human Immunodeficiency Virus (Topic) or Immunodeficiency Virus, Human (Topic) or Immunodeficiency Viruses, Human (Topic) or Virus, Human Immunodeficiency (Topic) or Viruses, Human Immunodeficiency (Topic) or Human Immunodeficiency Viruses (Topic) or Human T Cell Lymphotropic Virus Type III (Topic) or Human T-Cell Lymphotropic Virus Type III (Topic) or Human T-Cell Leukemia Virus Type III (Topic) or Human |

| | |
|---|---|
| | T Cell Leukemia Virus Type III (Topic) or LAV-HTLV-III (Topic) or Lymphadenopathy-Associated Virus (Topic) or Lymphadenopathy Associated Virus (Topic) or Lymphadenopathy-Associated Viruses (Topic) or Virus, Lymphadenopathy-Associated (Topic) or Viruses, Lymphadenopathy-Associated (Topic) or Human T Lymphotropic Virus Type III (Topic) or Human T-Lymphotropic Virus Type III (Topic) or AIDS Virus (Topic) or AIDS Viruses (Topic) or Virus, AIDS (Topic) or Viruses, AIDS (Topic) or Acquired Immune Deficiency Syndrome Virus (Topic) or Acquired Immunodeficiency Syndrome Virus (Topic) or HTLV-III (Topic) |
| #2 | Immunologic Deficiency Syndrome, Acquired (Topic) or Acquired Immune Deficiency Syndrome (Topic) or Acquired Immuno-Deficiency Syndrome (Topic) or Acquired Immuno Deficiency Syndrome (Topic) or Acquired Immuno-Deficiency Syndromes (Topic) or Immuno-Deficiency Syndrome, Acquired (Topic) or Immuno-Deficiency Syndromes, Acquired (Topic) or Syndrome, Acquired Immuno-Deficiency (Topic) or Syndromes, Acquired Immuno-Deficiency (Topic) or Immunodeficiency Syndrome, Acquired (Topic) or Acquired Immunodeficiency Syndromes (Topic) or Immunodeficiency Syndromes, Acquired (Topic) or Syndrome, Acquired Immunodeficiency (Topic) or Syndromes, Acquired Immunodeficiency (Topic) or AIDS (Topic) |
| #3 | #1 OR #2 |
| #4 | Mycobacterium, Atypical (Topic) or Tuberculoid Bacillus (Topic) or Atypical Mycobacterium (Topic) or Atypical Mycobacteria (Topic) or Mycobacteria, Atypical (Topic) or Nontuberculous Mycobacterium (Topic) or Non-Tuberculous Mycobacteria (Topic) or Mycobacterium szulgai (Topic) or Mycobacterium gordonae (Topic) or Mycobacterium duvalii (Topic) or Mycolicibacterium duvalii (Topic) or Mycobacterium flavescens (Topic) or Mycolicibacterium flavescens (Topic) or Mycobacterium gilvum (Topic) or Mycolicibacterium gilvum (Topic) or Mycobacterium obuense (Topic) or Mycolicibacterium obuense (Topic) or Mycobacterium terrae (Topic) or Mycolicibacter terrae (Topic) |
| #5 | #3 AND #4 |
| #6 | China (Topic) or Hong Kong (Topic) or Macau (Topic) or Taiwan (Topic) |
| #7 | #5 AND #6 |
| **Cochrane Library** | |
| #1 | MeSH descriptor: [HIV] explode all trees |
| #2 | (Human Immunodeficiency Virus) OR (Immunodeficiency Virus, Human) OR (Immunodeficiency Viruses, Human) OR (Virus, Human Immunodeficiency) OR (Viruses, Human Immunodeficiency) (Word variations have been searched) |
| #3 | (Human Immunodeficiency Viruses) OR (Human T Cell Lymphotropic Virus Type III) OR (Human T-Cell Lymphotropic Virus Type III) OR (Human T-Cell Leukemia Virus Type III) OR (Human T Cell Leukemia Virus Type III) (Word variations have been searched) |
| #4 | (LAV-HTLV-III) OR (Lymphadenopathy-Associated Virus) OR (Lymphadenopathy Associated Virus) OR (Lymphadenopathy-Associated Viruses) OR (Virus, Lymphadenopathy-Associated) (Word variations have been searched) |
| #5 | (Viruses, Lymphadenopathy-Associated) OR (Human T Lymphotropic Virus Type III) OR (Human T-Lymphotropic Virus Type III) OR (AIDS Virus) OR (AIDS Viruses) (Word variations have been searched) |
| #6 | (Virus, AIDS) OR (Viruses, AIDS) OR (Acquired Immune Deficiency Syndrome Virus) OR (Acquired Immunodeficiency Syndrome Virus) OR (HTLV-III) (Word variations have been searched) |
| #7 | #1 OR #2 OR #3 OR #4 OR #5 OR #6 |
| #8 | MeSH descriptor: [Acquired Immunodeficiency Syndrome] explode all trees |
| #9 | (Immunologic Deficiency Syndrome, Acquired) OR (Acquired Immune Deficiency Syndrome) OR (Acquired Immuno-Deficiency Syndrome) OR (Acquired Immuno Deficiency Syndrome) OR (Acquired Immuno-Deficiency Syndromes) (Word variations have been searched) |

| | |
|---|---|
| #10 | (Immuno-Deficiency Syndrome, Acquired) OR (Immuno-Deficiency Syndromes, Acquired) OR (Syndrome, Acquired Immuno-Deficiency) OR (Syndromes, Acquired Immuno-Deficiency) OR (Immunodeficiency Syndrome, Acquired) (Word variations have been searched) |
| #11 | (Acquired Immunodeficiency Syndromes) OR (Immunodeficiency Syndromes, Acquired) OR (Syndrome, Acquired Immunodeficiency) OR (Syndromes, Acquired Immunodeficiency) OR (AIDS) (Word variations have been searched) |
| #12 | #8 OR #9 OR #10 OR #11 |
| #13 | MeSH descriptor: [Nontuberculous Mycobacteria] explode all trees |
| #14 | (Mycobacterium, Atypical) OR (Tuberculoid Bacillus) OR (Atypical Mycobacterium) OR (Atypical Mycobacteria) OR (Mycobacteria, Atypical) (Word variations have been searched) |
| #15 | (Nontuberculous Mycobacterium) OR (Non-Tuberculous Mycobacteria) OR (Mycobacterium szulgai) OR (Mycobacterium gordonae) OR (Mycobacterium duvalii) (Word variations have been searched) |
| #16 | (Mycolicibacterium duvalii) OR (Mycobacterium flavescens) OR (Mycolicibacterium flavescens) OR (Mycobacterium gilvum) OR (Mycolicibacterium gilvum) (Word variations have been searched) |
| #17 | (Mycolicibacterium duvalii) OR (Mycobacterium flavescens) OR (Mycolicibacterium flavescens) OR (Mycobacterium gilvum) OR (Mycolicibacterium gilvum) (Word variations have been searched) |
| #18 | #13 OR #14 OR #15 OR #16 OR #17 |
| #19 | #7 OR #12 |
| #20 | MeSH descriptor: [China] explode all trees |
| #21 | MeSH descriptor: [Hong Kong] explode all trees |
| #22 | MeSH descriptor: [Macau] explode all trees |
| #23 | MeSH descriptor: [Taiwan] explode all trees |
| #24 | #20 OR #21 OR #22 OR #23 |
| #25 | #18 AND #19 AND #24 |
| **VIP Database** | |
| #1 | Title or Keywords = HIV OR human immunodeficiency virus OR human immunodeficiency viruses (In Chinese) |
| #2 | Title or Keywords = Acquired Immune Deficiency Syndrome (In Chinese) |
| #3 | #1 OR #2 |
| #4 | Title or Keywords = nontuberculous mycobacteria (In Chinese) |
| #5 | #3 AND #4 |
| **China National Knowledge Infrastructure** | |
| #1 | Topic = HIV OR human immunodeficiency virus OR human immunodeficiency viruses (In Chinese) |
| #2 | Topic = Acquired Immune Deficiency Syndrome (In Chinese) |
| #3 | #1 OR #2 |
| #4 | Topic = nontuberculous mycobacteria (In Chinese) |
| #5 | #3 AND #4 |
| **Wanfang Database** | |
| #1 | Topic = HIV OR human immunodeficiency virus OR human immunodeficiency viruses (In Chinese) |
| #2 | Topic = Acquired Immune Deficiency Syndrome (In Chinese) |
| #3 | #1 OR #2 |
| #4 | Topic = nontuberculous mycobacteria (In Chinese) |

| | |
|---|---|
| #5 | #3 AND #4 |
| **SinoMed** | |
| #1 | "HIV"[Mesh] (In Chinese) |
| #2 | Title/Abstract: Human Immunodeficiency Virus OR Lymphadenopathy Associated Virus OR AIDS Virus OR Acquired Immunodeficiency Syndrome Virus[Title/Abstract])) (In Chinese) |
| #3 | #1 OR #2 |
| #4 | "Acquired Immunodeficiency Syndrome"[Mesh] (In Chinese) |
| #5 | Title/Abstract: Acquired Immune Deficiency Syndrome OR Immunodeficiency Syndromes, Acquired OR AIDS (In Chinese) |
| #6 | #4 OR #5 |
| #7 | "Nontuberculous Mycobacteria"[Mesh] (In Chinese) |
| #8 | Mycobacterium, Atypical OR Nontuberculous Mycobacterium OR Mycobacterium szulgai OR Mycobacterium gordonae OR Mycobacterium duvalii OR Mycobacterium flavescens OR Mycobacterium gilvum OR Mycobacterium obuense OR Mycobacterium terrae (In Chinese) |
| #9 | #7 OR #8 |
| #10 | #3 OR #6 |
| #11 | #9 AND #10 |

CNKI – China National Knowledge Infrastructure

*VIP Database and CNKI both have synonym extension function, Wanfang Database has subject term expansion function. SinoMed, CNKI, Wanfang, and VIP Database were restricted to searching Chinese studies.

## Table S3. The Agency for Healthcare Research and Quality (AHRQ) Methodology Checklist for Cross-Sectional Study

| The Agency for Healthcare Research and Quality (AHRQ) Methodology Checklist for Cross-Sectional Study | | | |
|---|---|---|---|
| Major Components | Response options | | |
| 1. Define the source of information (survey, record review) | Yes | No | Unclear |
| 2. List inclusion and exclusion criteria for exposed and unexposed subjects (cases and controls) or refer to previous publications | Yes | No | Unclear |
| 3. Indicate time period used for identifying patients | Yes | No | Unclear |
| 4. Indicate whether or not subjects were consecutive if not population-based | Yes | No | Unclear |
| 5. Indicate if evaluators of subjective components of study were masked to other aspects of the status of the participants | Yes | No | Unclear |
| 6. Describe any assessments undertaken for quality assurance purposes (e.g., test/retest of primary outcome measurements) | Yes | No | Unclear |
| 7. Explain any patient exclusions from analysis | Yes | No | Unclear |
| 8. Describe how confounding was assessed and/or controlled | Yes | No | Unclear |
| 9. If applicable, explain how missing data were handled in the analysis | Yes | No | Unclear |
| 10. Summarize patient response rates and completeness of data collection | Yes | No | Unclear |
| 11. Clarify what follow-up, if any, was expected and the percentage of patients for which incomplete data or follow-up was obtained | Yes | No | Unclear |

**Table S4. The Newcastle-Ottawa Scale (NOS) for cohort study**

| The Newcastle-Ottawa Scale (NOS) for cohort study | |
|---|---|
| Major Components | Response options |
| Selection | |
| 1. Representativeness of the exposed cohort | |
| 1) truly representative of the average ________________ (describe) in the community | ☆ |
| 2) somewhat representative of the average _______________ in the community | ☆ |
| 3) selected group of users eg nurses, volunteers | / |
| 4) no description of the derivation of the cohort | / |
| 2. Selection of the non exposed cohort | |
| 1) drawn from the same community as the exposed cohort | ☆ |
| 2) drawn from a different source | / |
| 3) no description of the derivation of the non exposed cohort | / |
| 3. Ascertainment of exposure | |
| 1) secure record (eg surgical records) | ☆ |
| 2) structured interview □ | ☆ |
| 3) written self report | / |
| 4) no description | / |
| 4. Demonstration that outcome of interest was not present at start of study | |
| 1) yes | ☆ |
| 2) no | / |
| **Comparability*** | |
| 5. Comparability of cohorts on the basis of the design or analysis | |
| 1) study controls for ______________ (select the most important factor) | ☆ |
| 2) study controls for any additional factor (This criteria could be modified to indicate specific control for a second important factor.) | ☆ |
| **Outcome** | |
| 6. Assessment of outcome | |
| 1) independent blind assessment | ☆ |
| 2) record linkage | ☆ |

| | |
|---|---|
| 3) self report<br><br>4) no description | /<br><br>/ |
| 7. Was follow-up long enough for outcomes to occur<br><br>1) yes (select an adequate follow up period for outcome of interest)<br><br>2) no | <br><br>☆<br><br>/ |
| 8. Adequacy of follow up of cohorts<br><br>1) complete follow up - all subjects accounted for ¯<br><br>2) subjects lost to follow up unlikely to introduce bias - small number lost - > ____ % (select an adequate %)<br><br>follow up, or description provided of those lost)<br><br>3) follow up rate < ____% (select an adequate %) and no description of those lost<br><br>4) no statement | <br><br>☆<br><br>☆<br><br><br><br>/<br><br>/ |
| *, A study can be awarded a maximum of one star for each numbered item within the Selection and Exposure categories; a maximum of two stars can be given for Comparability. | |

**Table S5. The Newcastle-Ottawa Scale (NOS) for case-control study**

| The Newcastle-Ottawa Scale (NOS) for case-control study | |
|---|---|
| Major Components | Response options |
| Selection | |
| 1. Is the case definition adequate?<br>1) yes, with independent validation<br>2) yes, eg record linkage or based on self reports<br>3) no description | <br>☆<br>/<br>/ |
| 2. Representativeness of the cases<br>1) consecutive or obviously representative series of cases<br>2) potential for selection biases or not stated | <br>☆<br>/ |
| 3. Selection of Controls<br>1) community controls<br>2) hospital controls<br>3) no description | <br>☆<br>/<br>/ |
| 4. Definition of Controls<br>1) no history of disease (endpoint)<br>2) no description of source | <br>☆<br>/ |
| Comparability* | |
| 5. Comparability of cases and controls on the basis of the design or analysis<br>1) study controls for _______________ (Select the most important factor.)<br>2) study controls for any additional factor (This criteria could be modified to indicate specific control for a second important factor.) | <br>☆<br>☆ |
| Exposure | |
| 6. Ascertainment of exposure<br>1) secure record (eg surgical records)<br>2) structured interview where blind to case/control status<br>3) interview not blinded to case/control status<br>4) written self report or medical record only<br>5) no description | <br>☆<br>☆<br>/<br>/<br>/ |

| | |
|---|---|
| 7. Same method of ascertainment for cases and controls<br><br>1) yes<br><br>2) no | <br><br>☆<br><br>/ |
| 8. Non-Response rate<br><br>1) same rate for both groups<br><br>2) non respondents described<br><br>3) rate different and no designation | <br><br>☆<br><br>/<br><br>/ |
| *, A study can be awarded a maximum of one star for each numbered item within the Selection and Exposure categories; a maximum of two stars can be given for Comparability. | |

**Table S6. The Joanna Briggs Institute (JBI) Critical Appraisal Checklist for case series/case reports**

| The Joanna Briggs Institute (JBI) Critical Appraisal Checklist for case series/case reports | | | | |
|---|---|---|---|---|
| Major Components | Response options | | | |
| 1. Were patient's demographic characteristics clearly described? | Yes | No | Unclear | Not applicable |
| 2. Was the patient's history clearly described and presented as a timeline? | Yes | No | Unclear | Not applicable |
| 3. Was the current clinical condition of the patient on presentation clearly described? | Yes | No | Unclear | Not applicable |
| 4. Were diagnostic tests or assessment methods and the results clearly described? | Yes | No | Unclear | Not applicable |
| 5. Was the intervention(s) or treatment procedure(s) clearly described? | Yes | No | Unclear | Not applicable |
| 6. Was the post-intervention clinical condition clearly described? | Yes | No | Unclear | Not applicable |
| 7. Were adverse events (harms) or unanticipated events identified and described? | Yes | No | Unclear | Not applicable |
| 8. Does the case report provide takeaway lessons? | Yes | No | Unclear | Not applicable |
| Overall appraisal: Include ☐ Exclude ☐ Seek further info ☐ | | | | |

## Table S7. Quality assessment of the included studies*

| Study | Q1 | Q2 | Q3 | Q4 | Q5 | Q6 | Q7 | Q8 | Q9 | Q10 | Q11 | Total |
|---|---|---|---|---|---|---|---|---|---|---|---|---|
| The Joanna Briggs Institute (JBI) Critical Appraisal Checklist for case series/case reports | | | | | | | | | | | | |
| Song et al., 2011 | 1 | 0 | 1 | 1 | 0 | 0 | 0 | 1 | | | | 4 |
| The Agency for Healthcare Research and Quality (AHRQ) Methodology Checklist for Cross-Sectional Study | | | | | | | | | | | | |
| Ding et al., 2022 | 1 | 1 | 1 | 1 | 1 | 1 | 0 | 0 | 0 | 0 | 1 | 7 |
| Wang et al., 2017 | 1 | 1 | 1 | 1 | 1 | 1 | 0 | 0 | 0 | 0 | 1 | 7 |
| Wu et al., 2017 | 1 | 1 | 1 | 1 | 1 | 1 | 0 | 0 | 0 | 1 | 0 | 7 |
| Cao et al., 2021 | 1 | 1 | 1 | 1 | 1 | 1 | 0 | 0 | 0 | 1 | 0 | 7 |
| Jiang et al., 2014 | 1 | 0 | 1 | 1 | 1 | 1 | 0 | 0 | 0 | 0 | 0 | 5 |
| Meng et al., 2008 | 1 | 0 | 1 | 1 | 1 | 1 | 0 | 0 | 0 | 0 | 1 | 6 |
| Meng et al., 2018 | 1 | 1 | 1 | 1 | 1 | 1 | 0 | 0 | 0 | 0 | 1 | 7 |
| Yin et al., 2015 | 1 | 1 | 1 | 1 | 1 | 1 | 0 | 0 | 0 | 0 | 1 | 7 |
| Huang et al., 2022 | 1 | 1 | 1 | 1 | 1 | 1 | 0 | 0 | 0 | 1 | 0 | 7 |
| Zhou et al., 2013 | 1 | 0 | 1 | 1 | 1 | 1 | 0 | 0 | 0 | 0 | 0 | 5 |
| Wang et al., 2022 | 1 | 0 | 1 | 1 | 1 | 1 | 0 | 0 | 0 | 0 | 1 | 6 |
| Li et al., 2016 | 1 | 0 | 1 | 1 | 1 | 1 | 0 | 0 | 0 | 0 | 0 | 5 |
| Deng et al., 2013 | 1 | 0 | 1 | 1 | 1 | 1 | 0 | 0 | 0 | 0 | 0 | 5 |
| Wang et al., 2021 | 1 | 1 | 1 | 1 | 1 | 1 | 0 | 0 | 0 | 0 | 0 | 6 |
| Huang et al., 2021 | 1 | 0 | 1 | 1 | 1 | 1 | 0 | 0 | 0 | 0 | 0 | 5 |
| Li, 2018 | 1 | 0 | 1 | 1 | 1 | 1 | 0 | 0 | 0 | 0 | 0 | 5 |
| Zhu et al., 2013 | 1 | 1 | 1 | 1 | 1 | 1 | 0 | 0 | 0 | 0 | 0 | 6 |
| Sun et al., 2019 | 1 | 1 | 1 | 1 | 1 | 1 | 0 | 0 | 0 | 0 | 0 | 6 |
| Wang et al., 2019 | 1 | 1 | 1 | 1 | 1 | 1 | 0 | 0 | 0 | 1 | 0 | 7 |
| Zhang et al., 2021 | 1 | 1 | 1 | 1 | 1 | 1 | 0 | 0 | 0 | 0 | 0 | 6 |
| Li et al., 2018 | 1 | 1 | 1 | 1 | 1 | 1 | 0 | 0 | 0 | 1 | 0 | 7 |
| Liu et al., 2021 | 1 | 1 | 1 | 1 | 1 | 1 | 0 | 0 | 0 | 0 | 0 | 6 |
| Chou et al., 2011 | 1 | 1 | 1 | 1 | 1 | 1 | 0 | 0 | 0 | 0 | 0 | 6 |
| The Newcastle-Ottawa Scale (NOS) for cohort study and case-control study | | | | | | | | | | | | |

| | | | | | | | | | | | | |
|---|---|---|---|---|---|---|---|---|---|---|---|---|
| R. Lan et al., 2011 | 1 | 1 | 1 | 1 | 1 | 1 | 1 | 1 | | | | 8 |
| Zhang et al., 2011 | 1 | 1 | 1 | 1 | 0 | 1 | 1 | 0 | | | | 6 |
| Tian at al., 2022 | 1 | 1 | 1 | 1 | 1 | 1 | 1 | 1 | | | | 8 |
| Chiang et al., 2020 | 2 | 1 | 0 | 1 | 2 | 2 | 0 | 0 | | | | 8 |

*Specific numbers Q1-Q11 were shown in Tables S3 to S6 respectively.

**Table S8. Subgroup analyses of positive NTM isolates from patients with HIV/AIDS in different region***

| Observed indicators | Region | Number of included studies | Sample size | Heterogeneity | | MD or PR (95%CI) | Heterogeneity across subgroups | |
|---|---|---|---|---|---|---|---|---|
| | | | | P | $I^2$, % | | P | $I^2$, % |
| **Gender distribution (men)** | N | 3 | 109 | — | — | 0.881 (0.805, 0.943) | 0.222 | 5.7 |
| | S | 7 | 450 | 0.040 | 54.6 | 0.827 (0.765, 0.882) | | |
| | W | 5 | 266 | < 0.001 | 87.3 | 0.847 (0.699, 0.953) | | |
| | E | 6 | 711 | 0.001 | 75.6 | 0.923 (0.864, 0.968) | | |
| | C | 2 | 36 | — | — | 0.895 (0.762, 0.984) | | |
| **NTM species distribution** | | | | | | | | |
| *Mycobacterium avium* complex | N | 2 | 76 | — | — | 0.955 (0.909, 1.001) | < 0.001 | 121.3 |
| | S | 5 | 322 | 0.822 | 0.0 | 0.559 (0.505, 0.614) | | |
| | W | 3 | 193 | — | — | 0.633 (0.428, 0.839) | | |
| | E | 4 | 243 | < 0.001 | 97.3 | 0.639 (0.334, 0.943) | | |
| *Mycobacterium abscessus* complex | S | 4 | 311 | 0.778 | 0.0 | 0.029 (0.011, 0.053) | 0.442 | 3.0 |
| | W | 3 | 193 | — | — | 0.076 (0.030, 0.139) | | |
| | E | 3 | 141 | — | — | 0.024 (0.000, 0.115) | | |
| *Mycobacterium kansasii* | N | 1 | 71 | — | — | 0.042 (0.009, 0.119) | 0.222 | 2.7 |
| | S | 4 | 311 | 0.661 | 0.0 | 0.074 (0.046, 0.107) | | |
| | W | 3 | 193 | — | — | 0.139 (0.040, 0.282) | | |
| | E | 3 | 221 | — | — | 0.088 (0.002, 0.254) | | |
| *Mycobacterium gordonae* | S | 4 | 311 | — | 0.0 | 0.069 (0.041, 0.097) | 0.045 | 6.2 |
| | W | 2 | 134 | — | — | 0.029 (0.001, 0.057) | | |
| | E | 3 | 221 | — | — | 0.194 (0.009, 0.379) | | |
| Other NTM species† | N | 1 | 5 | — | — | 0.200 (0.036, 0.624) | < 0.001 | 28.9 |

| | | | | | | | | |
|---|---|---|---|---|---|---|---|---|
| | S | 5 | 322 | 0.842 | 0.0 | 0.262 (0.214, 0.310) | | |
| | W | 3 | 193 | — | — | 0.090 (0.024, 0.156) | | |
| | E | 4 | 243 | 0.150 | 43.5 | 0.091 (0.039, 0.142) | | |
| **Clinical symptoms** | | | | | | | | |
| Fever | N | 1 | 71 | — | — | 0.761 (0.650, 0.845) | 0.429 | 3.8 |
| | S | 7 | 331 | 0.003 | 69.2 | 0.729 (0.641, 0.818) | | |
| | W | 3 | 126 | — | — | 0.699 (0.578, 0.820) | | |
| | E | 2 | 116 | — | — | 0.652 (0.567, 0.737) | | |
| | C | 3 | 99 | — | — | 0.570 (0.280, 0.859) | | |
| Cough or expectoration | N | 1 | 71 | — | — | 0.338 (0.239, 0.454) | < 0.001 | 64.4 |
| | S | 6 | 318 | < 0.001 | 93.8 | 0.705 (0.527, 0.882) | | |
| | W | 3 | 126 | — | — | 0.884 (0.794, 0.974) | | |
| | E | 2 | 116 | — | — | 0.577 (0.489, 0.665) | | |
| | C | 2 | 90 | — | — | 0.520 (0.428, 0.611) | | |
| Dyspnea | N | 1 | 71 | — | — | 0.127 (0.068, 0.224) | < 0.001 | 25.7 |
| | S | 3 | 169 | — | — | 0.457 (0.131, 0.783) | | |
| | W | 2 | 103 | — | — | 0.403 (0.310, 0.497) | | |
| | C | 2 | 90 | — | — | 0.151 (0.080, 0.222) | | |
| Abdominal pain or diarrhea | N | 1 | 71 | — | — | 0.254 (0.167, 0.366) | 0.043 | 8.1 |
| | S | 3 | 169 | — | — | 0.341 (0.270, 0.412) | | |
| | W | 1 | 59 | — | — | 0.169 (0.095, 0.285) | | |
| | E | 2 | 116 | — | — | 0.276 (0.195, 0.357) | | |
| Night sweats | N | 1 | 71 | — | — | 0.085 (0.039, 0.172) | 0.016 | 12.2 |
| | S | 1 | 82 | — | — | 0.195 (0.124, 0.294) | | |
| | W | 1 | 23 | — | — | 0.391 (0.222, 0.592) | | |
| | E | 1 | 94 | — | — | 0.138 (0.083, 0.222) | | |

| | | | | | | | | |
|---|---|---|---|---|---|---|---|---|
| | C | 2 | 72 | — | — | 0.208 (0.114, 0.302) | | |
| Fatigue | N | 1 | 71 | — | — | 0.324 (0.227, 0.439) | 0.016 | 12.2 |
| | S | 5 | 282 | < 0.001 | 97.6 | 0.499 (0.199, 0.799) | | |
| | W | 1 | 59 | — | — | 0.153 (0.082, 0.265) | | |
| | E | 1 | 94 | — | — | 0.202 (0.133, 0.294) | | |
| | C | 2 | 90 | | | 0.322 (0.226, 0.419) | | |
| Weight loss | N | 1 | 71 | — | — | 0.141 (0.078, 0.240) | < 0.001 | 67.3 |
| | S | 5 | 282 | < 0.001 | 97.4 | 0.532 (0.231, 0.833) | | |
| | W | 2 | 82 | — | — | 0.602 (0.520, 0.683) | | |
| | E | 2 | 116 | — | — | 0.453 (0.363, 0.543) | | |
| | C | 2 | 72 | — | — | 0.305 (0.199, 0.412) | | |
| Superficial lymphadenectasis | S | 4 | 182 | 0.003 | 78.8 | 0.386 (0.224, 0.547) | 0.796 | 1.0 |
| | W | 2 | 73 | — | — | 0.472 (0.363, 0.581) | | |
| | E | 1 | 27 | — | — | 0.407 (0.245, 0.593) | | |
| | C | 3 | 99 | — | — | 0.388 (0.138, 0.637) | | |
| **Laboratory tests** | | | | | | | | |
| Hemoglobin count (g/L) | N | 2 | 104 | 0.001 | 91.7 | 85.823 (71.049, 100.597) | 0.605 | 1.0 |
| | S | 1 | 11 | — | — | 91.790 (72.744, 110.836) | | |
| | E | 2 | 191 | < 0.001 | 92.7 | 105.250 (68.672, 141.828) | | |
| $CD4^+$ T cell count (pieces/μL) | N | 1 | 33 | — | — | 112.320 (33.793, 190.847) | 0.001 | 16.6 |
| | S | 1 | 11 | — | — | 26.460 (5.907, 47.013) | | |
| | W | 2 | 73 | 0.048 | 74.4 | 53.836 (25.340, 82.333) | | |
| | E | 2 | 190 | 0.088 | 65.6 | 11.411 (8.379, 14.442) | | |
| Anemia | S | 3 | 103 | — | — | 0.404 (0.137, 0.671) | 0.011 | 9.1 |
| | W | 3 | 126 | — | — | 0.803 (0.711, 0.894) | | |
| | C | 1 | 9 | — | — | 0.556 (0.267, 0.811) | | |

| | | | | | | | | |
|---|---|---|---|---|---|---|---|---|
| CD4$^+$ T cell count ≤ 50 (pieces/μL) | N | 2 | 76 | — | — | 0.876 (0.779, 0.953) | 0.001 | 19.1 |
| | S | 4 | 182 | 0.344 | 9.8 | 0.763 (0.693, 0.827) | | |
| | W | 2 | 149 | — | — | 0.630 (0.550, 0.706) | | |
| | E | 2 | 124 | — | — | 0.672 (0.586, 0.754) | | |
| | C | 3 | 99 | — | — | 0.478 (0.235, 0.726) | | |
| CD4$^+$ T cell count 51-200 (pieces/μL) | N | 2 | 76 | — | — | 0.109 (0.037, 0.202) | 0.002 | 17.2 |
| | S | 4 | 182 | 0.545 | 0.0 | 0.190 (0.134, 0.251) | | |
| | W | 2 | 149 | — | — | 0.294 (0.223, 0.370) | | |
| | E | 2 | 124 | — | — | 0.187 (0.121, 0.262) | | |
| | C | 3 | 99 | — | — | 0.349 (0.254, 0.450) | | |
| CD4$^+$ T cell count > 200 (pieces/μL) | N | 2 | 76 | — | — | 0.000 (0.000, 0.028) | 0.016 | 12.2 |
| | S | 4 | 182 | 0.284 | 21.1 | 0.040 (0.010, 0.083) | | |
| | W | 2 | 149 | — | — | 0.058 (0.024, 0.103) | | |
| | E | 2 | 124 | — | — | 0.130 (0.074, 0.198) | | |
| | C | 3 | 99 | — | — | 0.101 (0.000, 0.394) | | |
| **Thoracic imaging manifestations** | | | | | | | | |
| Distribution of lesions | | | | | | | | |
| Single lung involvement | N | 1 | 71 | — | — | 0.127 (0.060, 0.227) | 0.024 | 9.4 |
| | S | 4 | 149 | 0.005 | 76.8 | 0.182 (0.044, 0.373) | | |
| | W | 1 | 50 | — | — | 0.160 (0.072, 0.291) | | |
| | E | 1 | 27 | — | — | 0.000 (0.000, 0.128) | | |
| Bilateral lung involvement | N | 1 | 71 | — | — | 0.761 (0.645, 0.854) | 0.002 | 14.6 |
| | S | 4 | 149 | < 0.001 | 85.6 | 0.786 (0.533,0.964) | | |
| | W | 1 | 50 | — | — | 0.840 (0.709, 0.928) | | |
| | E | 1 | 27 | — | — | 1.000 (0.872, 1.000) | | |
| No abnormalities | N | 2 | 104 | — | — | 0.156 (0.091, 0.234) | < 0.001 | 18.4 |

| | | | | | | | | |
|---|---|---|---|---|---|---|---|---|
| | S | 4 | 149 | 0.012 | 72.5 | 0.012 (0.000, 0.110) | | |
| | W | 1 | 50 | — | — | 0.000 (0.000, 0.071) | | |
| | E | 3 | 143 | — | — | 0.219 (0.000, 0.738) | | |
| Changes of lesion morphology and density | | | | | | | | |
| Patchy shadows | N | 1 | 71 | — | — | 0.423 (0.315, 0.538) | < 0.001 | 29.4 |
| | S | 3 | 141 | — | — | 0.498 (0.172, 0.824) | | |
| | W | 3 | 132 | — | — | 0.481 (0.306, 0.655) | | |
| | E | 1 | 27 | — | — | 0.074 (0.021, 0.234) | | |
| Nodules | N | 2 | 76 | — | — | 0.381 (0.273, 0.490) | 0.002 | 15.0 |
| | S | 3 | 141 | — | — | 0.360 (0.154, 0.567) | | |
| | W | 3 | 132 | — | — | 0.453 (0.240, 0.665) | | |
| | E | 2 | 121 | — | — | 0.182 (0.121, 0.243) | | |
| Millet shadow | N | 2 | 104 | — | — | 0.036 (0.006, 0.085) | 0.791 | 1.0 |
| | S | 1 | 31 | — | — | 0.065 (0.008, 0.214) | | |
| | W | 2 | 109 | — | — | 0.067 (0.025, 0.125) | | |
| | E | 3 | 143 | — | — | 0.030 (0.000, 0.195) | | |
| Cavitary lesion | N | 3 | 109 | — | — | 0.007 (0.000, 0.078) | 0.001 | 18.6 |
| | S | 5 | 206 | < 0.001 | 90.7 | 0.221 (0.052, 0.455) | | |
| | W | 2 | 109 | — | — | 0.137 (0.077, 0.210) | | |
| | E | 3 | 143 | — | — | 0.061 (0.006, 0.154) | | |
| | C | 1 | 35 | — | — | 0.343 (0.191, 0.522) | | |
| Stripe shadow | N | 2 | 104 | — | — | 0.400 (0.307, 0.497) | 0.033 | 8.7 |
| | S | 2 | 110 | — | — | 0.512 (0.415, 0.608) | | |
| | W | 1 | 50 | — | — | 0.620 (0.472, 0.753) | | |
| | E | 1 | 27 | — | — | 0.630 (0.424, 0.806) | | |
| Bronchiectasis | N | 1 | 5 | — | — | 0.200 (0.036, 0.624) | 0.018 | 11.9 |

| | | | | | | | | |
|---|---|---|---|---|---|---|---|---|
| | S | 2 | 110 | — | — | 0.354 (0.265, 0.443) | | |
| | W | 1 | 50 | — | — | 0.340 (0.224, 0.478) | | |
| | E | 1 | 27 | — | — | 0.333 (0.186, 0.522) | | |
| | C | 1 | 27 | — | — | 0.667 (0.478, 0.814) | | |
| Accompanying Signs | | | | | | | | |
| Thoracic lymph node enlargement | N | 2 | 76 | — | — | 0.762 (0.648, 0.861) | < 0.001 | 18.8 |
| | S | 4 | 170 | < 0.001 | 95.4 | 0.316 (0.030, 0.710) | | |
| | W | 2 | 73 | — | — | 0.590 (0.473, 0.702) | | |
| | E | 1 | 27 | — | — | 0.296 (0.138, 0.502) | | |
| Hydropericardium | N | 1 | 71 | — | — | 0.141 (0.078, 0.240) | 0.464 | 1.5 |
| | S | 2 | 60 | — | — | 0.079 (0.011, 0.147) | | |
| | W | 2 | 82 | — | — | 0.127 (0.056, 0.197) | | |
| Hydrothorax | N | 1 | 71 | — | — | 0.211 (0.123, 0.324) | < 0.001 | 26.1 |
| | S | 2 | 60 | — | — | 0.170 (0.082, 0.279) | | |
| | W | 3 | 132 | — | — | 0.228 (0.019, 0.554) | | |
| | E | 3 | 143 | — | — | 0.015 (0.000, 0.047) | | |
| | C | 1 | 35 | — | — | 0.114 (0.032, 0.267) | | |
| Pleural thickening | N | 2 | 76 | — | — | 0.275 (0.175, 0.375) | 0.023 | 11.4 |
| | S | 2 | 110 | — | — | 0.149 (0.084, 0.215) | | |
| | W | 1 | 50 | — | — | 0.080 (0.032, 0.188) | | |
| | E | 1 | 27 | — | — | 0.074 (0.021, 0.234) | | |
| | C | 1 | 27 | — | — | 0.111 (0.039, 0.281) | | |
| **Treatment outcome** | | | | | | | | |
| Symptoms improve‡ | N | 2 | 104 | — | — | 0.732 (0.649, 0.814) | 0.202 | 3.2 |
| | S | 3 | 162 | — | — | 0.619 (0.397, 0.841) | | |
| | C | 1 | 9 | — | — | 0.889 (0.565, 0.980) | | |

| | | | | | | | | |
|---|---|---|---|---|---|---|---|---|
| Death | N | 2 | 104 | — | — | 0.036 (0.005, 0.084) | 0.136 | 4.0 |
| | S | 3 | 162 | — | — | 0.096 (0.053, 0.148) | | |
| | C | 1 | 9 | — | — | 0.000 (0.000, 0.336) | | |
| Others§ | N | 2 | 104 | — | — | 0.200 (0.127, 0.274) | 0.580 | 1.1 |
| | S | 3 | 162 | — | — | 0.270 (0.048, 0.491) | | |
| | C | 1 | 9 | — | — | 0.111 (0.020, 0.435) | | |

MD – mean deviation, PR – prevalence rate, CI – confidence interval, NTM – nontuberculous mycobacterial, W – western region, E – eastern region, N – northern region, S – southern region, C – central region

*Subgroup analyses were not performed for the number of included studies less than 5. We performed the subgroup analysis using the metaprop functional module, which does not show heterogeneity for the number of included studies less than or equal to 3 per subgroup.

†All other NTM species accounted for less than the above four species.

‡Symptom improve is defined as getting better after treatment during hospitalization.

§Others include automatic discharge, transfer to another hospital, and no apparent improvement.

**Table S9. Subgroup analyses of positive NTM isolates from patients with HIV/AIDS in sample size per study***

| Observed indicators | Sample size per study | Number of included studies | Sample size | Heterogeneity | | MD or PR (95%CI) | Heterogeneity across subgroups | |
|---|---|---|---|---|---|---|---|---|
| | | | | P | $I^2$, % | | P | $I^2$, % |
| **Gender distribution (men)** | < 50 | 13 | 328 | 0.002 | 61.7 | 0.869 (0.797, 0.930) | 0.656 | 0.2 |
| | ⩾50 | 10 | 1244 | < 0.001 | 84.0 | 0.873 (0.819, 0.918) | | |
| **NTM species distribution** | | | | | | | | |
| *Mycobacterium avium* complex | < 50 | 7 | 178 | < 0.001 | 88.4 | 0.632 (0.444, 0.820) | 0.881 | 0.0 |
| | ⩾50 | 7 | 656 | < 0.001 | 97.1 | 0.652 (0.473, 0.830) | | |
| *Mycobacterium abscessus* complex | < 50 | 5 | 162 | 0.288 | 20.0 | 0.047 (0.013, 0.095) | 0.540 | 0.4 |
| | ⩾50 | 5 | 483 | 0.026 | 63.7 | 0.040 (0.014, 0.076) | | |
| *Mycobacterium kansasii* | < 50 | 4 | 140 | 0.143 | 44.7 | 0.065 (0.016, 0.136) | 0.402 | 0.7 |
| | ⩾50 | 7 | 656 | < 0.001 | 82.4 | 0.109 (0.057, 0.174) | | |
| *Mycobacterium gordonae* | < 50 | 4 | 140 | 0.118 | 48.9 | 0.074 (0.015, 0.133) | 0.646 | 0.2 |
| | ⩾50 | 5 | 526 | < 0.001 | 87.8 | 0.094 (0.033, 0.154) | | |
| Other NTM species† | < 50 | 7 | 178 | 0.014 | 62.4 | 0.201 (0.106, 0.296) | 0.260 | 1.3 |
| | ⩾50 | 6 | 585 | < 0.001 | 86.2 | 0.134 (0.066, 0.202) | | |
| **Clinical symptoms** | | | | | | | | |
| Fever | < 50 | 10 | 277 | 0.139 | 33.6 | 0.746 (0.683, 0.808) | 0.045 | 4.0 |
| | ⩾50 | 6 | 466 | < 0.001 | 85.1 | 0.616 (0.506, 0.727) | | |
| Cough or expectoration | < 50 | 8 | 255 | < 0.001 | 88.5 | 0.714 (0.578, 0.851) | 0.476 | 0.5 |
| | ⩾50 | 6 | 466 | < 0.001 | 97.7 | 0.617 (0.387, 0.847) | | |
| Dyspnea | < 50 | 4 | 143 | 0.044 | 62.9 | 0.374 (0.247, 0.502) | 0.773 | 0.1 |
| | ⩾50 | 4 | 290 | < 0.001 | 97.8 | 0.327 (0.031, 0.622) | | |
| Abdominal pain or diarrhea | < 50 | 3 | 94 | — | — | 0.297 (0.204, 0.389) | 0.590 | 0.3 |
| | ⩾50 | 4 | 321 | 0.035 | 65.1 | 0.268 (0.220, 0.316) | | |

| | | | | | | | | |
|---|---|---|---|---|---|---|---|---|
| Night sweats | < 50 | 2 | 32 | — | — | 0.332 (0.171, 0.493) | 0.034 | 4.5 |
| | ⩾50 | 4 | 310 | 0.107 | 50.9 | 0.148 (0.092, 0.204) | | |
| Fatigue | < 50 | 4 | 130 | < 0.001 | 98.0 | 0.462 (0.028, 0.897) | 0.561 | 0.3 |
| | ⩾50 | 6 | 466 | < 0.001 | 90.6 | 0.328 (0.196, 0.459) | | |
| Weight loss | < 50 | 6 | 157 | < 0.001 | 97.2 | 0.498 (0.153, 0.844) | 0.671 | 0.6 |
| | ⩾50 | 6 | 466 | < 0.001 | 95.1 | 0.413 (0.229, 0.597) | | |
| Superficial lymphadenectasis | < 50 | 7 | 171 | < 0.001 | 78.8 | 0.469 (0.313, 0.625) | 0.149 | 2.1 |
| | ⩾50 | 3 | 210 | — | — | 0.315 (0.177, 0.454) | | |
| **Laboratory tests** | | | | | | | | |
| Hemoglobin count (g/L) | < 50 | 3 | 66 | < 0.001 | 87.9 | 103.553 (82.393, 124.714) | 0.073 | 3.2 |
| | ⩾50 | 2 | 240 | 0.002 | 89.2 | 82.807 (74.567, 91.046) | | |
| $CD4^+$ T cell count (pieces/μL) | < 50 | 4 | 89 | < 0.001 | 86.7 | 42.930 (12.320, 73.540) | 0.463 | 0.5 |
| | ⩾50 | 2 | 218 | < 0.001 | 99.8 | 26.371 (5.498, 58.240) | | |
| Anemia | < 50 | 6 | 179 | < 0.001 | 89.7 | 0.539 (0.328, 0.750) | 0.005 | 7.8 |
| | ⩾50 | 1 | 59 | — | — | 0.864 (0.755, 0.930) | | |
| $CD4^+$ T cell count ≤ 50 (pieces/μL) | < 50 | 7 | 176 | < 0.001 | 80.7 | 0.764 (0.590, 0.906) | 0.357 | 0.9 |
| | ⩾50 | 6 | 454 | < 0.001 | 93.0 | 0.644 (0.469, 0.801) | | |
| $CD4^+$ T cell count 51-200 (pieces/μL) | < 50 | 7 | 176 | < 0.001 | 78.1 | 0.208 (0.081, 0.368) | 0.803 | 0.1 |
| | ⩾50 | 6 | 454 | < 0.001 | 80.4 | 0.244 (0.159, 0.340) | | |
| $CD4^+$ T cell count > 200 (pieces/μL) | < 50 | 7 | 176 | 0.563 | 0.0 | 0.006 (0.000, 0.033) | 0.068 | 3.3 |
| | ⩾50 | 6 | 454 | < 0.001 | 89.8 | 0.094 (0.025, 0.197) | | |
| **Thoracic imaging manifestations** | | | | | | | | |
| Distribution of lesions | | | | | | | | |
| Single lung involvement | < 50 | 4 | 79 | < 0.001 | 87.4 | 0.120 (0.000, 0.413) | 0.961 | 0.0 |
| | ⩾50 | 3 | 218 | — | — | 0.142 (0.097, 0.192) | | |
| Bilateral lung involvement | < 50 | 4 | 79 | < 0.001 | 91.0 | 0.852 (0.486, 1.000) | 0.921 | 0.0 |

| | | | | | | | | |
|---|---|---|---|---|---|---|---|---|
| | ⩾50 | 3 | 218 | — | — | 0.822 (0.760, 0.877) | | |
| No abnormalities | < 50 | 6 | 134 | 0.003 | 72.6 | 0.075 (0.004, 0.196) | 0.875 | 0.0 |
| | ⩾50 | 4 | 312 | < 0.001 | 98.3 | 0.112 (0.000, 0.489) | | |
| Changes of lesion morphology and density | | | | | | | | |
| Patchy shadows | < 50 | 4 | 94 | < 0.001 | 96.4 | 0.471 (0.061, 0.880) | 0.699 | 0.2 |
| | ⩾50 | 4 | 277 | 0.769 | 0.0 | 0.389 (0.332, 0.446) | | |
| Nodules | < 50 | 5 | 99 | 0.081 | 51.8 | 0.508 (0.364, 0.652) | 0.095 | 2.8 |
| | ⩾50 | 5 | 371 | < 0.001 | 92.8 | 0.324 (0.164, 0.484) | | |
| Millet shadow | < 50 | 4 | 113 | 0.025 | 67.8 | 0.080 (0.009, 0.198) | 0.275 | 1.2 |
| | ⩾50 | 4 | 274 | < 0.001 | 88.7 | 0.025 (0.000, 0.116) | | |
| Cavitary lesion | < 50 | 9 | 231 | < 0.001 | 75.2 | 0.110 (0.033, 0.216) | 0.730 | 0.1 |
| | ⩾50 | 5 | 371 | < 0.001 | 95.5 | 0.160 (0.025, 0.373) | | |
| Stripe shadow | < 50 | 3 | 73 | — | — | 0.783 (0.499, 0.975) | 0.042 | 4.1 |
| | ⩾50 | 3 | 218 | — | — | 0.440 (0.276, 0.611) | | |
| Bronchiectasis | < 50 | 4 | 72 | 0.015 | 71.4 | 0.399 (0.188, 0.611) | 0.691 | 0.2 |
| | ⩾50 | 2 | 147 | — | — | 0.354 (0.276, 0.431) | | |
| Accompanying Signs | | | | | | | | |
| Thoracic lymph node enlargement | < 50 | 6 | 128 | < 0.001 | 88.2 | 0.533 (0.266, 0.791) | 0.698 | 0.2 |
| | ⩾50 | 3 | 218 | — | — | 0.423 (0.050, 0.860) | | |
| Hydropericardium | < 50 | 3 | 83 | — | — | 0.102 (0.038, 0.166) | 0.674 | 0.2 |
| | ⩾50 | 2 | 130 | — | — | 0.120 (0.065, 0.176) | | |
| Hydrothorax | < 50 | 6 | 167 | < 0.001 | 87.2 | 0.146 (0.024, 0.331) | 0.530 | 0.4 |
| | ⩾50 | 4 | 274 | < 0.001 | 86.3 | 0.095 (0.020, 0.213) | | |
| Pleural thickening | < 50 | 4 | 72 | 0.181 | 38.5 | 0.138 (0.032, 0.243) | 0.777 | 0.1 |
| | ⩾50 | 3 | 218 | — | — | 0.159 (0.057, 0.261) | | |

| | | | | | | | | |
|---|---|---|---|---|---|---|---|---|
| **Treatment outcome** | | | | | | | | |
| Symptoms improve‡ | < 50 | 4 | 107 | < 0.001 | 89.2 | 0.721 (0.487, 0.956) | 0.668 | 0.2 |
| | ⩾50 | 2 | 168 | — | — | 0.668 (0.597, 0.739) | | |
| Death | < 50 | 4 | 107 | 0.589 | 0.0 | 0.074 (0.026, 0.138) | 0.430 | 0.6 |
| | ⩾50 | 2 | 168 | — | — | 0.062 (0.029, 0.105) | | |
| Others§ | < 50 | 4 | 107 | < 0.001 | 89.5 | 0.201 (0.004, 0.398) | 0.580 | 0.3 |
| | ⩾50 | 2 | 168 | — | — | 0.260 (0.194, 0.326) | | |

MD – mean deviation, PR – prevalence rate, CI – confidence interval, NTM – nontuberculous mycobacterial

*Subgroup analyses were not performed for the number of included studies less than 5. We performed the subgroup analysis using the metaprop functional module, which does not show heterogeneity for the number of included studies less than or equal to 3 per subgroup.

†All other NTM species accounted for less than the above four species.

‡Symptom improve is defined as getting better after treatment during hospitalization.

§Others include automatic discharge, transfer to another hospital, and no apparent improvement.
